# Structural, optical and luminescence properties of Fe^3+^-doped mixed alkali zirconia-borate glasses for warm orange-red photonic applications

**DOI:** 10.1038/s41598-026-45270-1

**Published:** 2026-03-26

**Authors:** D. Vinay, C. Devaraja, Utpal Deka, R. S. Gedam

**Affiliations:** 1 Department of Physics, Manipal Institute of Technology Bengaluru, Manipal Academy of Higher Education, Manipal, Karnataka 576104 India; 2 Department of Physics, Sikkim Manipal Institute of Technology, Sikkim Manipal University (SMU), Majitar, India; 3 Department of Physics, Visvesvaraya National Institute of Technology, Nagpur, Maharashtra 440010 India

**Keywords:** Fe^3+^-doped borate glasses, Melt-quenching technique, Optical bandgap and Urbach energy, Nonlinear optical properties, Orange-red photoluminescence, Materials science, Optics and photonics, Physics

## Abstract

A novel series of Fe^3+^ doped mixed alkali zirconia-borate glasses with the composition 60B_2_O_3_–25Na_2_O–10Li_2_O–(5 − x)ZrO_2_–xFe_2_O_3_ (x = 0–1 mol%) were synthesized using the melt-quenching technique. The novelty of this work lies in combining the structural modifications with enhanced optical properties and orange-red luminescence in Fe^3+^ doped mixed alkali zirconia-borate glasses for warm photonic applications. X-ray diffraction confirmed the non-crystalline nature of glass, while SEM images and EDS spectra were utilised for morphological and elemental analysis. The density of the glasses decreased from 2.4197 to 2.4168 g cm^−3^ before gradually increasing to 2.4324 g cm^−3^. FTIR and Raman spectral studies showed the formation of non-bridging orthoborate units from pentaborate and di-pentaborate units. The optical absorption band at 450 nm is associated with the Fe^3+^ transition ^6^A_1g_ (^6^S) → ^4^A_1g_ (^4^G); ^4^E_g_ (^4^G). The decrease in direct bandgap from 3.90 to 3.10 eV and indirect bandgap from 3.44 to 2.77 eV, along with an increase in Urbach energy from 0.245 to 0.273 eV, indicates the disorder in structure. An increase in refractive index leads to an increase in third-order susceptibility and nonlinear refractive index. The photoluminescence spectra exhibited orange-red emission at (^4^T_2g_(^4^G) → ^6^A_1g_(^6^S)) with 550 nm, 560 nm and 570 nm excitation wavelengths. The CIE chromaticity and CCT values show that Fe^3+^ doped glasses are suitable for warm-emitting orange-red photonic applications.

## Introduction

Advanced glass materials are developing as a major emphasis in material science due to their diverse structural and optical features, which allow for a wide range of industrial and technological applications. Among these materials, glasses have become ubiquitous and flexible because of their inherent benefits, which include easy doping with different elements for modifying their properties, cost-effectiveness in fabrication, and simple processing methods^1,2^. In modern applications, the potential of an assortment of glass-forming systems, including borosilicate^3,4^, tellurite^5^, silicate^6^, phosphate^6^, boro-tellurite^7^, vanadate^8^, and chalcohalide glasses^9^, has been extensively investigated. Borate-based glasses have drawn a lot of interest in the field since their recent developments, like smart lighting technologies, and next-generation display devices, due to their distinctive arrangements in the structure, low melting points, non-toxicity, thermal and chemical stability^10,11^. Energy-saving devices^12^, laser media^13^, thermal and UV sensing, optoelectronic modulators^11^, and photonic applications^14,15^ are a few recent advancements in these glasses.

The distinct structure of B_2_O_3_ in the glass network substantially determines the special features of the borate glasses. The two coordination states of B_2_O_3_ are BO_3_ (trigonal) units and BO_4_ (tetrahedral) units, which will link to form the boroxol rings (B_3_O_6_), and other structures like metaborate, pyroborate, and other structures. These linkages of BO_3_ and BO_4_ units form bridging oxygens (BOs) in the glassy network. Inclusion of alkali oxides like Li_2_O, Na_2_O, into the glass structures initially forms BOs. Furthermore, high concentrations of alkali oxides act as modifiers by breaking the network, forming non-bridging oxygens (NBOs) in the network, and this process is called the borate anomaly^16–18^. Thus, the modification of the network will directly impact on structural, physical, and optical properties of the glasses due to the variation in the ratio of BOs to NBOs units in the network of the glass^19,20^.

Transition metal oxides in borate glass act as dopants, modifying the structural and optical properties for advancements in electro-optical devices. ZrO_2_ with the + 4-oxidation state contributes to the glass in network formation with [ZrO_4_] and [ZrO_6_] units^21^. The presence of ZrO_2_ in the glass matrix can improve the fragility and thermal stability of the glass^21,22^. Similarly, Fe_2_O_3_ with various oxidation states, Fe^3+^ (ferric) and Fe^2+^ (ferrous), exhibits [FeO_4_] and [FeO_6_] units with tetrahedral and octahedral domains where Fe^3+^ occupies both the domain and Fe^2+^ occupies only in octahedral domain^23^. The stable state of Fe_2_O_3_ is the Fe^3+^ (trivalent) state, which develops a spin-forbidden transition with a weak absorption band near 400–500 nm. The presence of an ionic state improves the ligand field; in addition, it illuminates the colour and improves the optical features of the host glass^24^. The illumination in luminescence of the Fe_2_O_3_-doped glass can change to various regions in the CIE colour coordination as the glass is converted into glass ceramics^25^.

Huang et al.^25^ studied the effect of Fe_2_O_3_ doped in magnesium alumino-silicate-based glasses. The addition of Fe_2_O_3_ showed an increase in NBOs. Optical spectra reveal the presence of Fe^2+^ and Fe^3+^ in glass and an increase in the ionic nature of the glass. Photoluminescence spectra showed dark blue emission due to Fe^3+^ transitions in glass. Pattar et al.^26^ examined the iron oxide-doped sodium boro-tellurite glasses. The existence of Fe^3+^ was shown in EPR spectra, and the formation of BO_3_ units from BO_4_ units was recorded. Optical properties were calculated and compared with the change in structure in the glass network. Xu et al.^27^ discussed the impact of radioactive nuclear waste Fe_2_O_3_ in Mo-borosilicate glass. Homogeneity in the structure was seen above 3.85 mol%, and the addition of Fe_2_O_3_ contributes towards network formation. The incorporation of iron oxides improved the chemical durability of the sample.

### Problem statement

The ability of Fe^3+^ doped borate glasses to alter the optical and structural characteristics of the glass network has gained significant interest. Incorporating Fe^3+^ into mixed alkali zirconia borate glasses can alter borate structural units and create localised electronic states that impact optical absorption and luminescence. Fe^3+^ doped mixed alkali zirconia borate glasses have promising characteristics for electro-optical and photonic applications. Understanding the role of Fe^3+^ ions in influencing the structural and optical properties of this glass system is an essential research problem.

### Research gap

Although Fe^3+^ based borate glasses have been thoroughly examined for their structural and optical properties, Fe^3+^-doped mixed alkali zirconia-borate glasses are comparatively unexplored, particularly for their orange-red luminescence and non-linear optical response from the optical bandgap. The relationship between structural modification of borate units and the consequent optical and luminescence properties has not been extensively explored. Moreover, limited investigations have studied the influence of Fe^3+^ inclusion on nonlinear optical characteristics such as nonlinear refractive index and third-order susceptibility in these glass systems. Therefore, a detailed investigation linking structural modifications and optical luminescent properties is necessary to evaluate their potential for photonic applications.

### Current research focus

In agreement with earlier literatures, mixed alkali zirconia borate glasses doped with Fe_2_O_3_ are not explored much with orange-red emission, electro-optical and photonic applications. The current study focused on novel glass systems with a matrix of 60B_2_O_3_–25Na_2_O–10Li_2_O–(5 − x) ZrO_2_–xFe_2_O_3_, with x = 0.0–1.0 mol%, synthesised while taking into consideration the abovementioned challenges. The physical, structural, optical and photoluminescence characteristics were investigated for potential applications in electro-optical and photonic applications.

## Experimental details

### Sample preparation

**Fig. 1:**
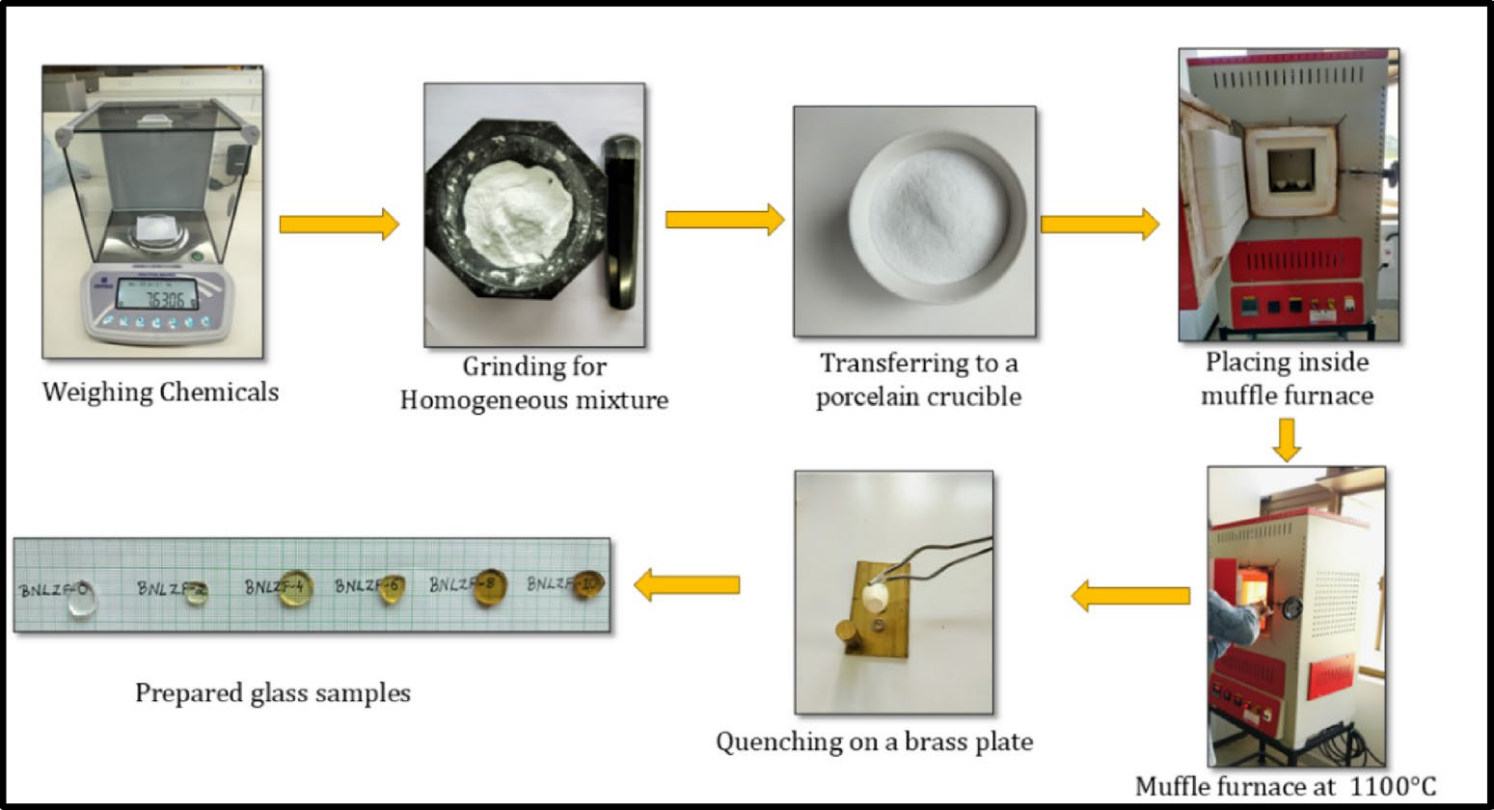
Synthesis of BNLZF glasses by the melt quenching technique.

The novel glass series with the composition 60B_2_O_3_–25Na_2_O–10Li_2_O–(5 − x)ZrO_2_–xFe_2_O_3_, x = 0.0, 0.2, 0.4, 0.6, 0.8, 1.0 mol%, was synthesised using the melt quenching technique. The synthesised glass system is coded as BNLZF-00, BNLZF-02, BNLZF-04, BNLZF-06, BNLZF-08, and BNLZF-10 and tabulated in Table 1. The inorganic and basic ingredients are composed of boric acid, sodium carbonate, lithium carbonate, zirconium carbonate, and ferric sulphate from Sigma Aldrich, with 99% purity, with AR grade. The chemicals were weighed precisely according to the stoichiometric ratio, and all the chemicals were grinded in an agate mortar and pestle for about 30 min to achieve homogeneity in the mixture. The well-grinded mixture is filled inside the porcelain crucible and placed in the muffle furnace. The temperature is set for 1100 °C and held for 30 min. The melt in the crucible is stirred to ensure homogeneity and quenched on the brass rectangular plate, which consists of steel rings placed on it and is pressed with the brass mould on the super-cooled liquid. The synthesis procedure is illustrated in Fig. 1. The prepared glass samples were stored in the desiccator to prevent airborne moisture to the samples. Furthermore, the samples were crushed to get the powder, and round pellet-shaped samples were polished with sandpaper graded from 320 to 2000 microns to perform further characterisations.

**Table 1:** Chemical composition of the glass.

Sample code	B_2_O_3_ mol%	Na_2_O mol%	Li_2_O mol%	ZrO_2_ mol%	Fe_2_O_3_ mol%
Sample composition					
BNLZF-00	60	25	10	5.0	0.0
BNLZF-02	60	25	10	4.8	0.2
BNLZF-04	60	25	10	4.6	0.4
BNLZF-06	60	25	10	4.4	0.6
BNLZF-08	60	25	10	4.2	0.8
BNLZF-10	60	25	10	4.0	1.0

### Sample characterization

The X-ray diffraction (XRD) analysis of the powder sample was carried out using an XRD RIGAKU equipped with a 6000-type diffractometer with a Cu radiation source (λ = 1.541 Å). The instrument was operated at the tube voltage of 40 kV and a tube current of 30 mA. The diffraction data were collected over a 2θ range of 10°–80° at a scanning rate of 4° per minute. The morphological and elemental configuration of the glass samples is examined using the ZEISS EVO 10 scanning electron microscope (SEM), which has an element energy dispersive spectroscopy (EDS) system with an additional backscattered electron detection. To minimize the effects of the charges generated by the beam of an electron, a thin layer of Au sputtering was required to coat on the sample surface. An ATIR-FTIR spectrometer is used to record the infrared absorption spectra of fine powdered samples at room temperature, ranging from 400 to 1600 cm^−1^. A confocal Raman microscope of Horiba Jobin Yvon-Xplora plus V2.1 multiline is scanned in the range from 50 to 1600 cm^−1^ at room temperature to obtain the Raman spectra. The resulting absorption spectra are deconvoluted to resolve the peaks. The deconvolution of FTIR spectra and Raman spectra, band fitting was carried out using an ancillary software “Origin 2024b”, with a “Gaussian fit” of deconvolution in a built-in function “Multiple Peak fit” of absorption spectra for prepared samples. The R^2^ (fitting parameter) values were found to be 0.99, which is equal to 1, with the error rate of ± 0.001. The density of the synthesised samples was ascertained using Archimedes’ buoyancy effect at room temperature, with distilled water as the suspension medium. Using a Shimadzu UV-1900I model spectrometer, UV–Vis absorption spectra were obtained at room temperature. The refractive index of the glass was measured using a Gemology Gemstone Gem Refractometer using a Monochromatic Light Filter with a minimum gradation value: 0.01 RI. The photoluminescence spectra are obtained using Horiba Jobin Yvon using the FL-1039/40 model with a 200–900 nm wavelength range at room temperature, with an excitation wavelength of 425 nm and emission wavelength of 600 nm.

## Results and discussion

### X-ray diffraction (XRD)

**Fig. 2:**
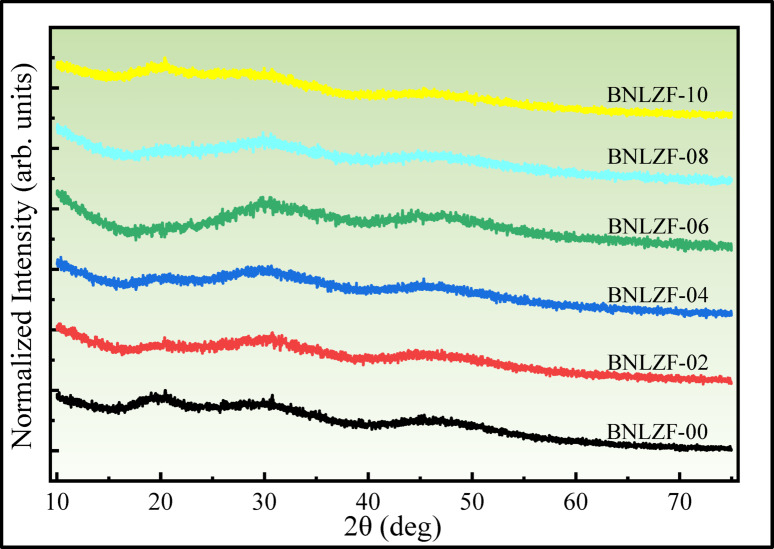
XRD spectra of BNLZF glasses.

The XRD patterns of the BNLZF glass series are displayed in Fig. 2. The scanning angle 2θ (deg) ranges from 10° to 80°, and the absence of sharp peaks indicates the non-crystalline nature of the glass structure. The broad bands observed at 15°–25°, and 25°–40° are due to the presence of Li^+^ and Na^+^ alkali ions in the borate structure, creating short-range disordered structures, and the 40°–55° broad hump with low intensity is due to the presence of ZrO_2_ with the borate structure. These broad humps indicate the short-range disorders in the glassy network^28–30^.

### SEM and EDS

**Fig. 3:**
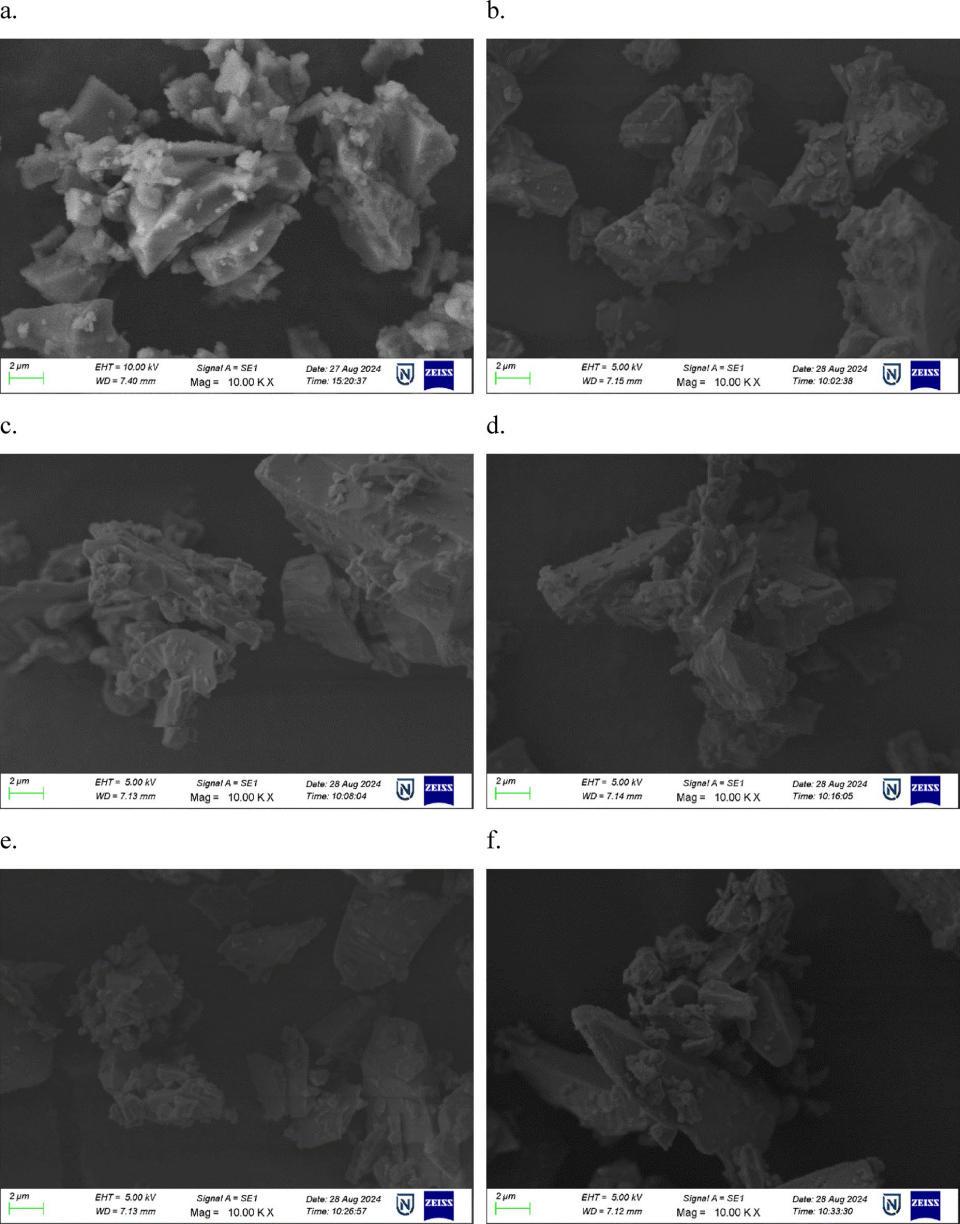
(**a**–**f**) SEM images of BNLZF glasses.

**Fig. 4:**
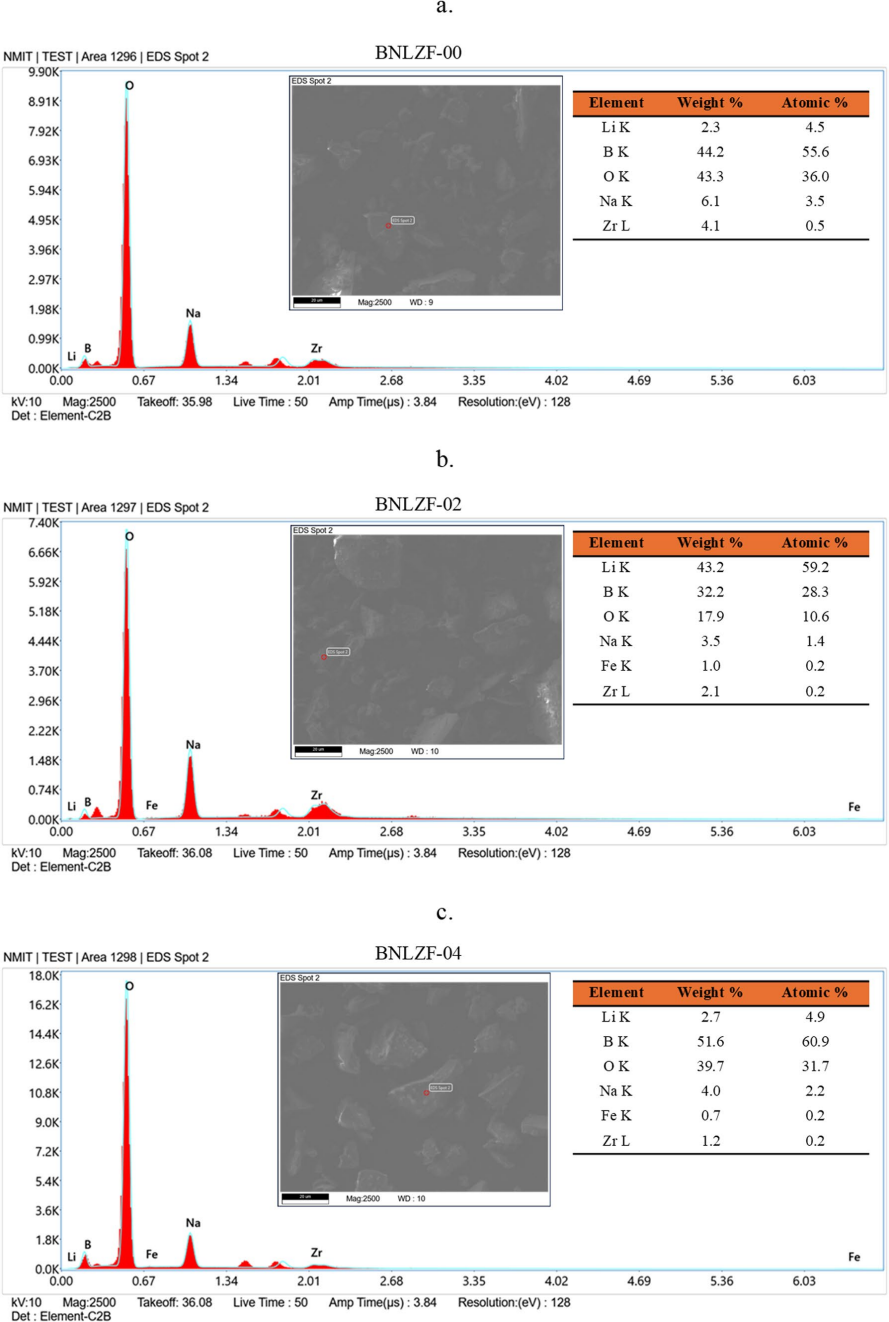

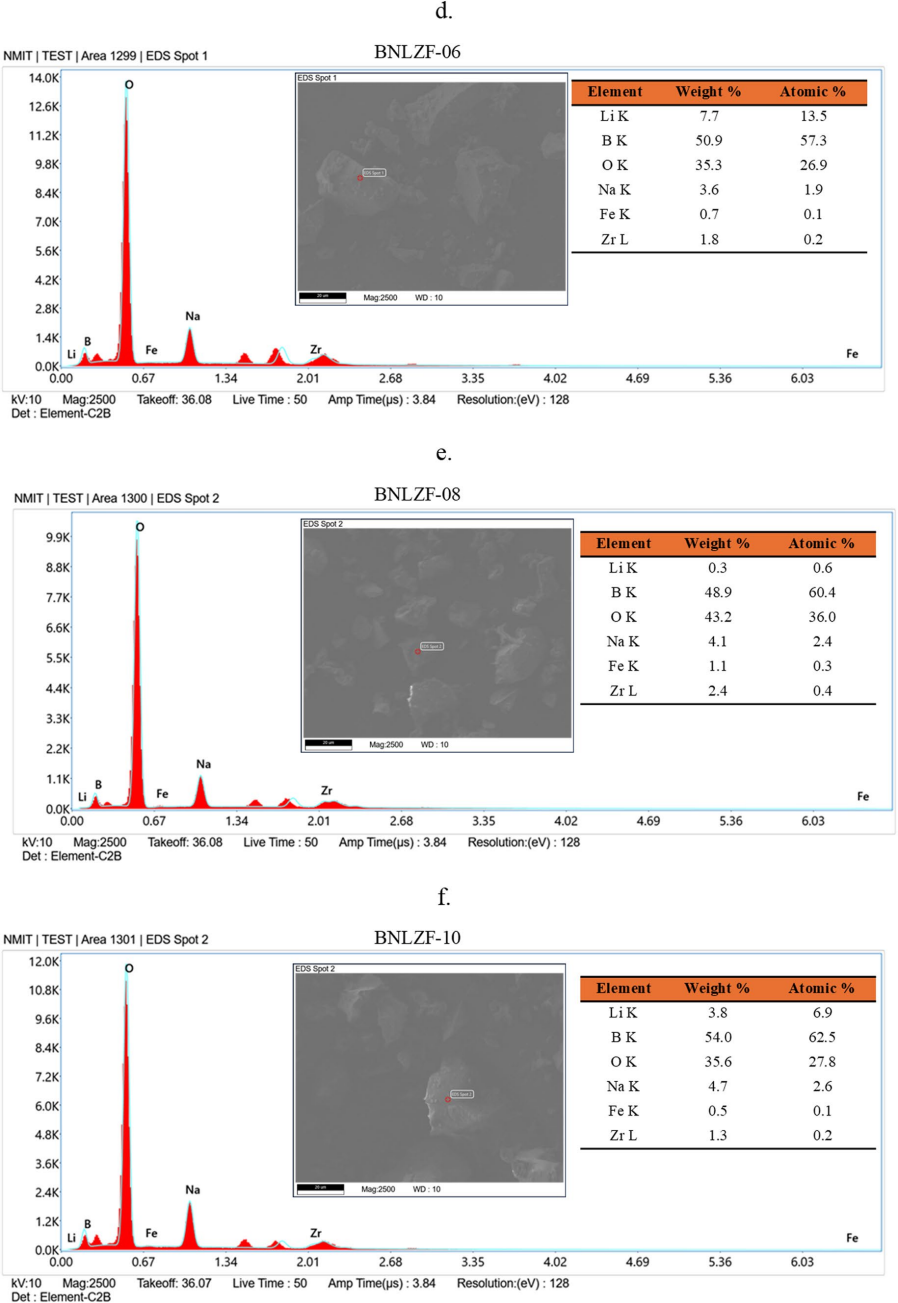
(**a**–**f**) EDS spectra of BNLZF glasses.

To obtain morphological analysis on the surface level and find the composition of elements is crucial for material characterisation by using SEM images and EDS spectra. Figure 3 shows the images of SEM of the BNLZF glasses, where the particles were subjected to a high vacuum and magnified at a 10.00 KX level. Powder of the glass is noticeable in the images as small, tiny particles amidst a specific size in a few hundred nm ranges. It is feasible to detect amorphous nature in the chemical configurations on these nanometric dust particles that lack an ordered crystal structure in 10.00 KX magnification. The EDS spectra of the BNLZF glass series are shown in Fig. 4. The increase in the peak of Fe is seen as the concentration of Fe_2_O_3_ has been introduced from BNLZF-02 to BNLZF-10 glass samples. The presence of B, Na, Li, Zr, Fe and O peaks confirms the occurrence of the elements in the glass structure. Nevertheless, based on the region that was measured, the weight percentage and the atomic percentage values in the data gathered from the spot of EDS spectra do not show a regular distribution. This further demonstrates that the structure is not entirely homogeneous^31^. The gold sputtering process on glass samples caused an additional peak between 1.34 and 2.01, representing the presence of Au. The lack of foreign elements indicated in the spectra provides the information of no extraneous materials from the crucible were contained in the glass samples^32–35^.

### Physical properties

The physical properties of BNLZF glasses, including molar volume (${V}_{M}$), molar volume of boron (${V}_{m}^{B}$), average boron-boron distance (${d}_{{<}B{-}B{>}}$), oxygen molar volume (${V}_{O}$), oxygen packing density ($OPD$), transition metal ion (TMI) concentration (${N}_{Fe}$), inter-ionic distance (${r}_{Fe}$), polaron radius (${r}_{p}$), and field strength ($F$) was calculated based on the molecular weight (${M}_{W}$) and density ($\rho$) values of glass samples. The corresponding calculated values were listed in Table 2.

**Table 2:** The physical properties of the BNLZF glasses (All the parameters are calculated with an error range of ± 0.001).

Glass code	BNLZF-00	BNLZF-02	BNLZF-04	BNLZF-06	BNLZF-08	BNLZF-10
${M}_{W}$, g mol^−1^	115.7943	116.5978	117.4014	118.2050	119.0085	119.8121
$\rho$, g cm^−3^	2.4197	2.4168	2.4203	2.4235	2.4271	2.4324
${V}_{M}$, cm^3^ mol^−1^	47.8548	48.2447	48.5070	48.7745	49.0332	49.2567
${V}_{O}^{B}$, × 10^−5^ m^3^ mol^−1^	5.9819	6.0306	6.0634	6.0968	6.1292	6.1571
${d}_{{<}B{-}B{>}}$, Å	4.6313	4.6438	4.6522	4.6607	4.6690	4.6760
${V}_{O}$, cm^3^	21.2866	21.4230	21.5204	21.6199	21.7153	21.7950
$OPD$, g atom l^−1^	47.0172	46.6787	46.4676	46.2537	46.0504	45.8820
${N}_{Fe}$, × 10^20^ ions cm^−3^	0	0.7489	1.4898	2.2224	2.9476	3.6677
${r}_{Fe}$, nm	0	2.3724	1.8864	1.6509	1.5026	1.3970
${r}_{p}$, Å	0	9.5620	7.6031	6.6541	6.0563	5.6307
$F$, cm^−2^	0	3.2812	5.1897	6.7756	8.1791	9.4623

Density is a vital characteristic that can be used effectively to examine variations in structures within the glass system. It functions as an essential measurement of the internal structures and atomic packing of a material. The degree of compactness, the size of interstitial voids, the geometrical structures of structural units, and the individual atoms’ coordination number are key variables in determining the glass density. In this work, the density of the glass samples was evaluated using a laboratory setup built on Archimedes’ principle. Equation (1) is used to measure the density of the glass samples and is tabulated in Table 2.

1 $$\rho =\frac{{w}_{a}}{{w}_{a}-{w}_{d}}{\rho }_{d}$$

where ${\rho }_{d}$ is the density of the distilled water = 1.0 g cm^−3^, ${w}_{a}$ is the weight of the glass samples in air, and ${w}_{d}$ is the weight of the glass samples in liquid^36^.

The ${V}_{M}$ of the glass is determined using Eq. (2). The computed values are displayed in Table 2.

2 $${V}_{m}=\frac{{M}_{w}}{\rho }$$

3 $${V}_{m}^{B}=\frac{{V}_{m}}{2(1-{X}_{B})}$$

4 $${d}_{{<}B{-}B{>} }={\left(\frac{{V}_{m}^{B}}{{N}_{A}}\right)}^\frac{1}{3}$$

5 $${V}_{O}=\frac{{V}_{m}}{\sum_{i}{(x{n}_{0})}_{i}}$$

6 $$OPD=\frac{1000\times \sum_{i}{(x{n}_{0})}_{i}}{{V}_{m}}$$

7 $${N}_{Fe}=\frac{{N}_{A} \times mol{\% }of cation \times valance \; of \; cation}{{V}_{m}}$$

8 $${r}_{Fe}={\left(\frac{1}{{N}_{Fe}}\right)}^{1/3}$$

9 $${r}_{p}=\frac{1}{2}{\left(\frac{\pi }{{6N}_{Fe}}\right)}^\frac{1}{3}$$

10 $$F=\frac{Z}{{({{\boldsymbol{r}}}_{{\boldsymbol{p}}})}^{2}}$$

where ${M}_{w}$ is the molecular weight of BNLZF glasses, ${X}_{B}$ is B_2_O_3_ mol% in BNLZF glasses, $x{n}_{0}$ is the mole fraction of the composition with the number of oxygens in the mole fraction, ${N}_{A}$ is Avogadro’s number, $Z$ valency of cation doped in the BNLZF glasses^36–38^.

**Fig. 5:**
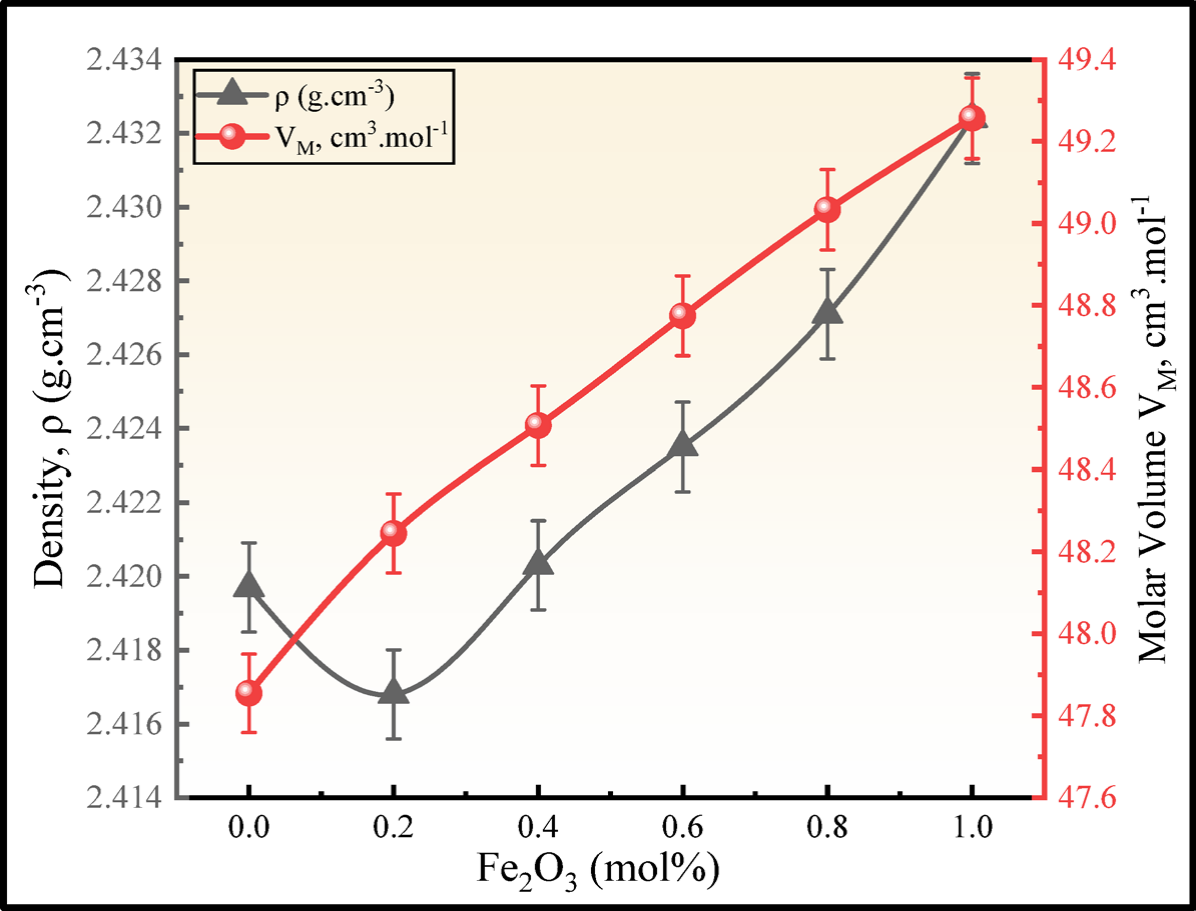
Density and molar volume plot of the BNLZF glasses.

Figure 5 shows the variation in the density and molar volume of all BNLZF glasses. As $\rho$ increases from 2.4197 to 2.4324 g cm^−3^ the ${V}_{m}$ also increases from 47.8548 to 49.2568 cm^3^ mol^−1^ along with it, as shown in Table 2. At 0.2 mol% of Fe_2_O_3_ replacing ZrO_2_, $\rho$ reduces to 2.4168 g cm^−3^ due to the presence of Fe^2+^ and Fe^3+^ with the ionic radii of 0.077 nm and 0.064 nm, respectively^39^, in the tetrahedral and octahedral sites inside the glass network between the small amount of zirconium and the large amount of borate structures. Furthermore, the higher molecular weight of the Fe_2_O_3_ (159.69 g mol^−1^) compared to the molecular weight of the ZrO_2_ (123.218 g mol^−1^) leads to an increase in $\rho$. ${V}_{m}$ behaves in a similar pattern to that of $\rho$, which can be presumed with the following explanations: (1) the increase in oxygen number, where Fe_2_O_3_ has a greater number of oxygens than ZrO_2_. (2) The ${V}_{m}$ of Fe_2_O_3_ (41.52 cm^3^ mol^−1^) is greater than ${V}_{m}$ of ZrO_2_ (20.849 cm^3^ mol^−1^). (3) Formation of NBOs as described in the FTIR and Raman section^39,40^.

${V}_{O}^{B}$ has increased from 5.9819 × 10^−5^ to 6.1571 × 10^−5^ m^3^ mol^−1^, and an increase in ${d}_{{<}B{-}B{>}}$ from 4.6313 to 4.6760 Å as the concentration of the iron is replaced with zirconium. This results in the replacement of Fe^3+^ ions, which has increased the boron distance in the glass network^39^.

**Fig. 6:**
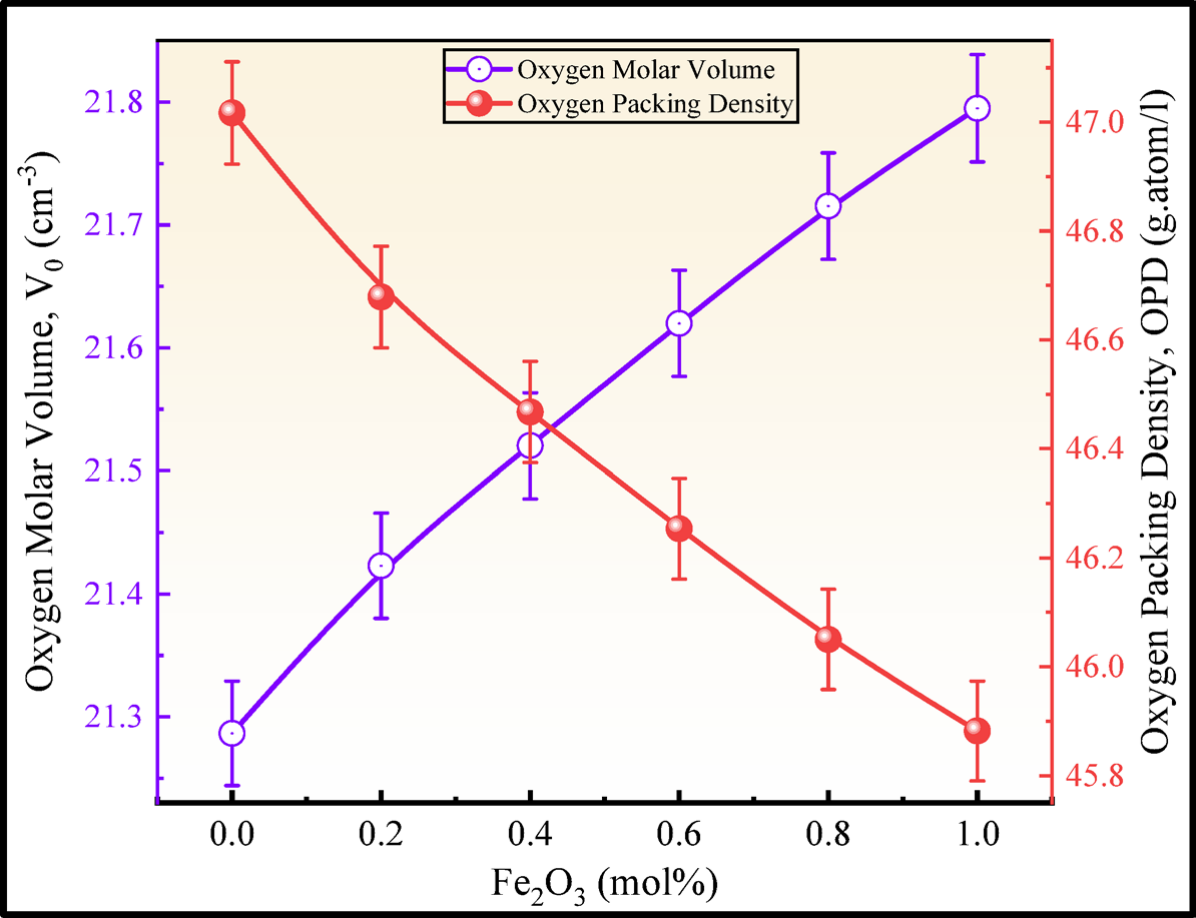
${V}_{O}$ and $OPD$ plot of the BNLZF glasses.

The ${V}_{O}$ has increased since the trend of $OPD$ is in the reverse direction was recorded as seen in Fig. 6. Therefore, the addition of iron oxide has been shown to increase in NBOs within the molecular structure of the network of the glass. The ${V}_{O}$ in the glass structure has increased from 21.2866 to 21.7950 cm^3^ and $OPD$ decreased to 45.8820 g atom l^−1^ from 47.0172 g atom l^−1^. The addition of Fe_2_O_3_ had a sensitive effect upon the way the oxygen molecules inhabited the vacant space inside the molecular structure of the glass network, causing this change in $OPD$. These two physical characteristics are known to be related to the quantity of oxygen atoms adorned within the molecular structure of the glass network. In the molecular structure of the glass, the oxygen has a larger ionic radius than the other components (O^2−^: 0.140 nm, B^3+^: 0.020 nm, Na^+^: 0.102 nm, Li^+^: 0.076 nm, Zr^4+^: 0.080 nm, Fe^2+^: 0.077 nm, and Fe^3+^: 0.064 nm). This evidence indicates that the addition of the Fe^3+^ affects both the molar and free volumes of the glassy network^41^.

**Fig. 7:**
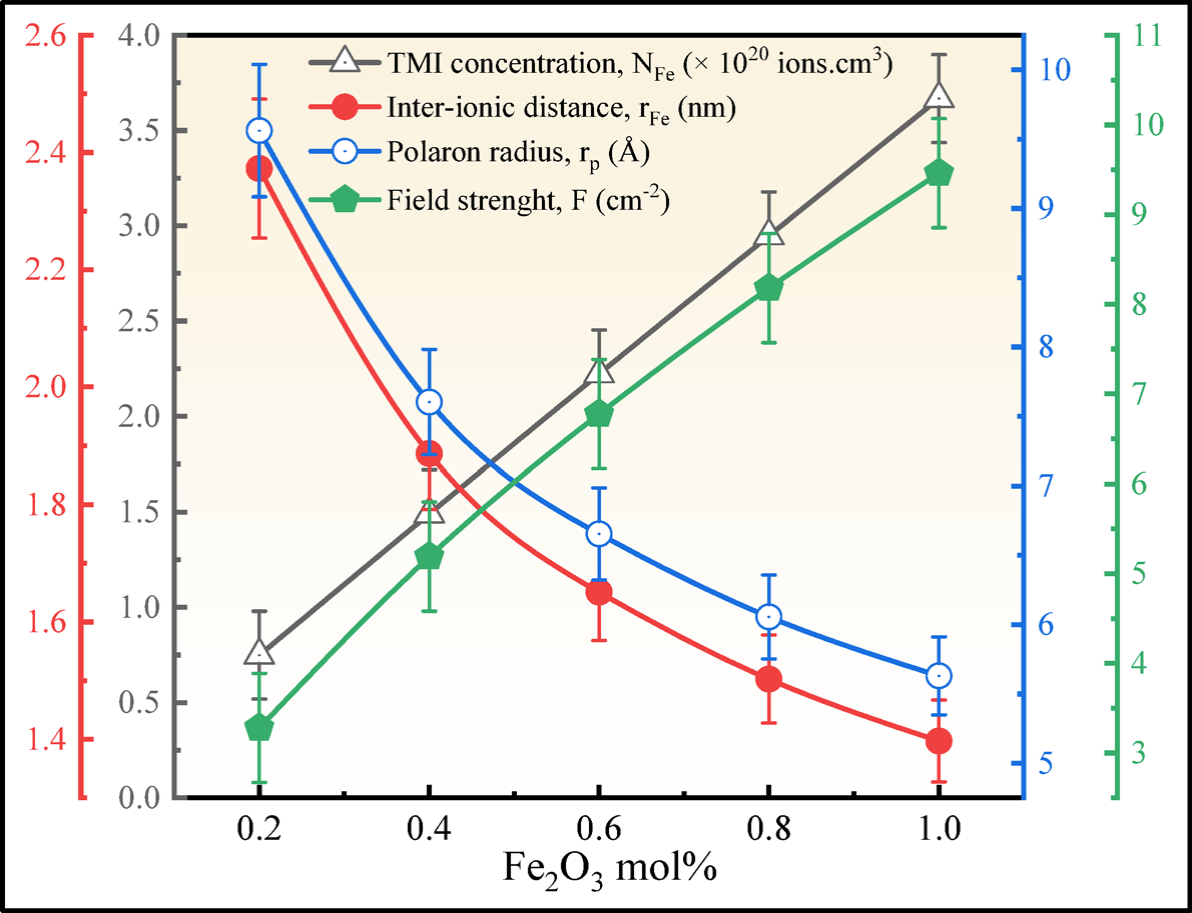
Comparison of ${N}_{Fe}$, ${r}_{Fe}$, ${r}_{p}$ and $F$ plot of the BNLZF glasses.

Furthermore, an increase in the concentration of Fe_2_O_3_, the ionic concentration of Fe^3+^ (${N}_{Fe}$) increased from 0.7489 × 10^20^ to 3.6677 × 10^20^ ions/cm^3^ in the glass structure. Furthermore, field strength has increased from 3.2812 to 9.4623 cm^−2^, enhancing the exerted electrostatic force from the Fe^3+^ ions on the surrounding oxygen ions in the glass network. However, the inter-ionic distance (${r}_{Fe}$) and polaron radius (${r}_{p}$) increased from 2.3724 to 1.3970 nm and 9.5620–5.6307 Å, respectively, as shown in Fig. 7. This reduces the distance between the Fe^3+^ ions and improves the distortion in the glass by the localised charge carriers within the glassy network^26,40^.

### Fourier transform infra-red (FTIR) spectroscopy analysis

**Fig. 8:**
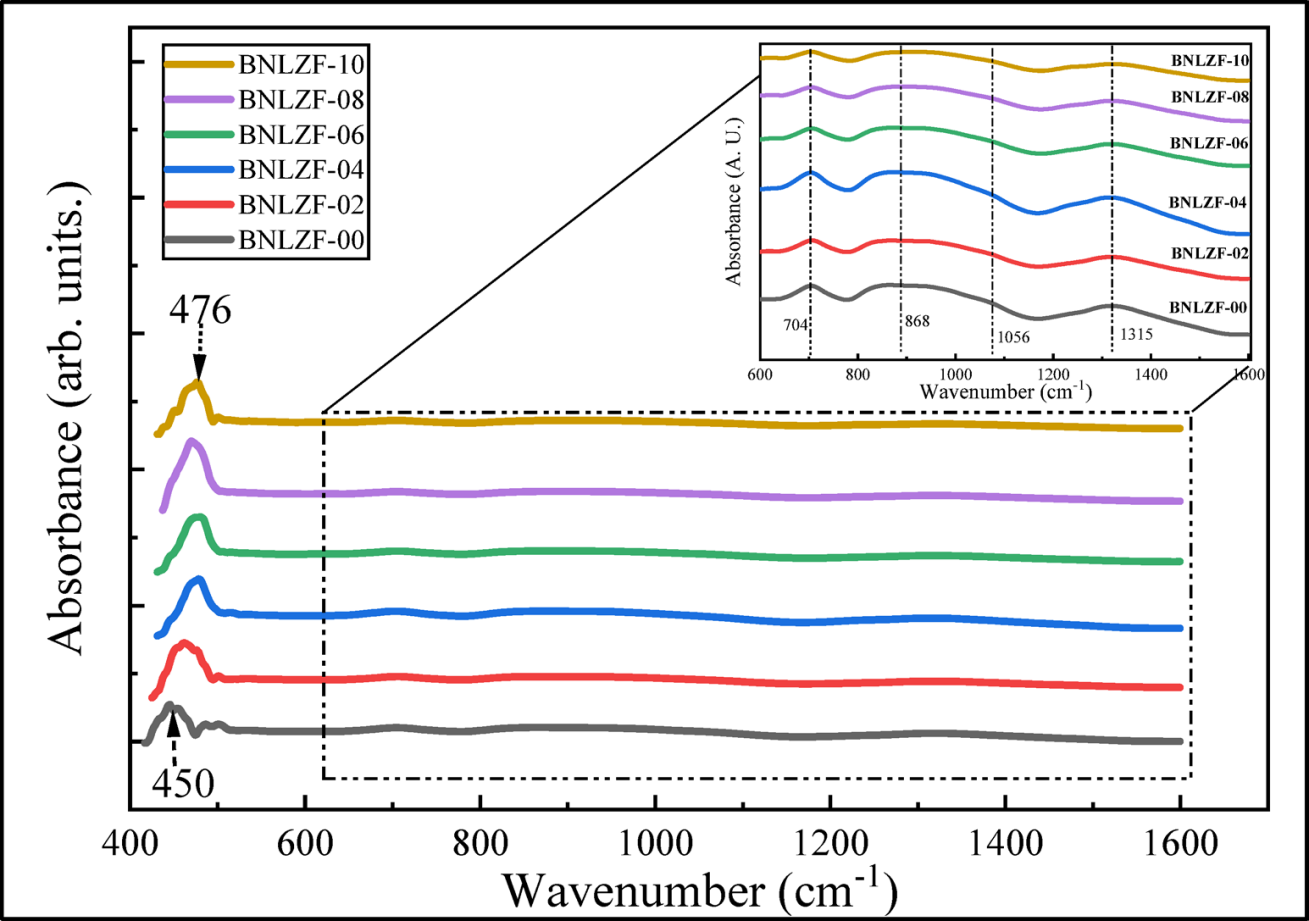
FTIR spectra of BNLZF glasses.

**Fig. 9:**
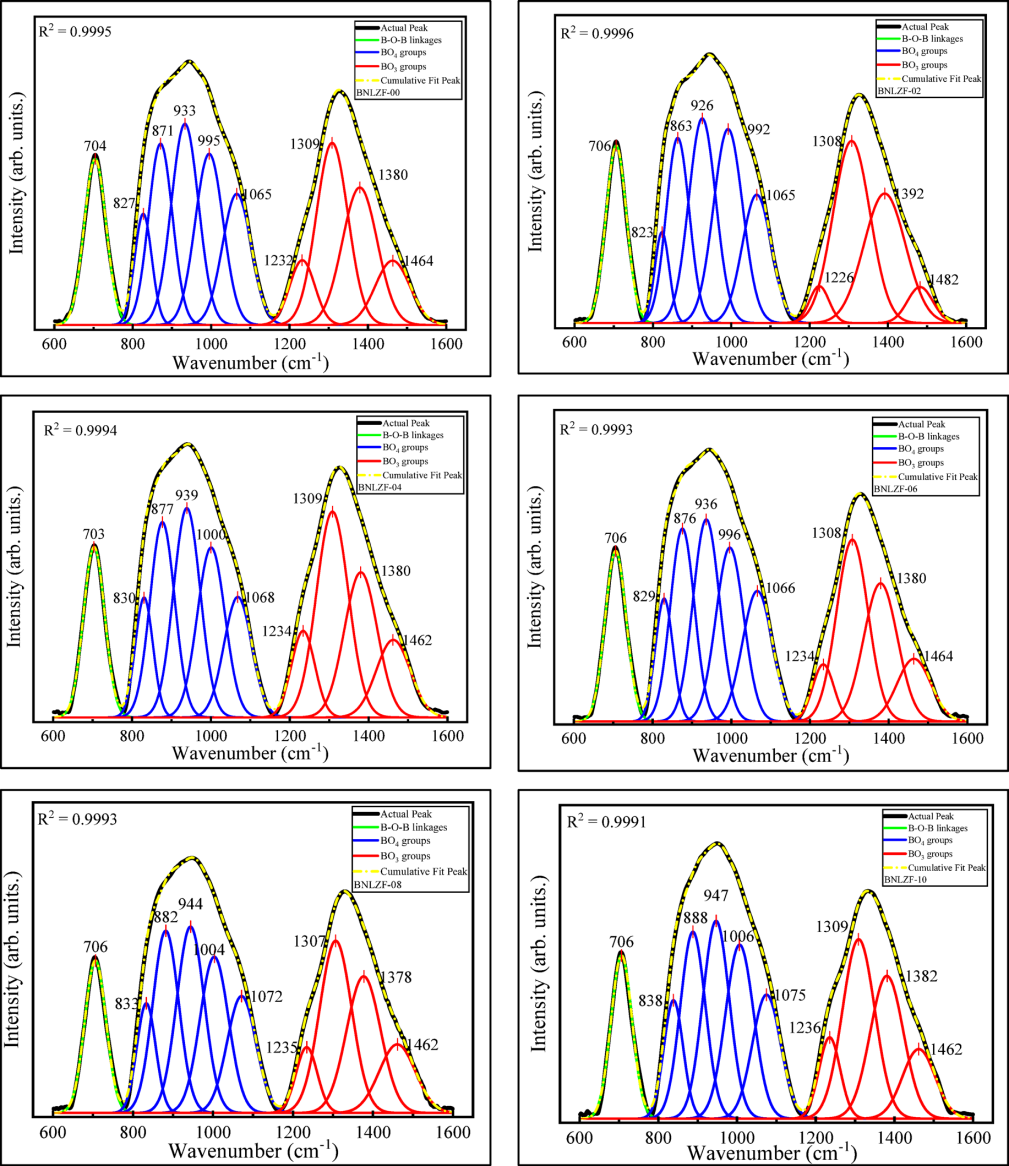
Deconvoluted FTIR spectra of BNLZF glasses.

FTIR spectroscopy is categorized as vibrational spectroscopy, and it is a sensitive and non-destructive method for examining the arrangements of the structural components in the matrix of the glass system. FTIR absorption spectra indicate numerous peaks of BNLZF glasses in distinct vibrations of the elements in the glass matrix, including B_2_O_3_, Na_2_O, Li_2_O, ZrO_2_, and Fe_2_O_3_. Figure 8 displays the FTIR spectra of the synthesized glasses in the range of 400–1600 cm^−1^. As seen in the figure, the spectra contain four regions: (1) 400–600 cm^−1^ attributed to metallic cations vibration, (2) 600–800 cm^−1^ accredited to B–O–B and Zr–O–Zr bending vibrations, (3) 800–1200 cm^−1^ is ascribed to symmetrical and asymmetrical vibrations of BO_4_ units, and (4) 1200–1600 cm^−1^ is endorsed to asymmetrical vibrations of BO_3_ units^42,43^. Table 3 lists the detected bands and their related band assignments. The deconvoluted spectra are displayed in Fig. 9.

**Table 3:** Band assignments of deconvoluted FTIR spectra of BNLZF glasses.

BNLZF-00	BNLZF-02	BNLZF-04	BNLZF-06	BNLZF-08	BNLZF-10	Band assignments	Refs.
704	706	703	706	706	706	Bending vibrations of B–O–B linkages with Zr–O–Zr linkages in ZrO_4_ units. Fe–O bonds in FeO_4_ units	^34,44,45^
827	823	830	829	833	838	B–O–B bending of tri-, tetra- and penta-borate units	^45,46^
871	863	877	876	882	888		
933	926	939	936	944	947	Diborate units [B_4_O_7_^2−^] of BO_4_ or [BØ_4_]^−^ groups	^46–48^
995	992	1000	996	1004	1006		
1065	1065	1068	1066	1076	1075	Stretching vibrations of tri-, tetra- and penta-borate groups	^49^
1232	1226	1234	1234	1235	1236	BO_3_^3−^ (ortho-borate) units	^46^
1309	1308	1309	1308	1307	1309	B_2_O_5_^4−^ (pyro-borate dimers)	^50^
1380	1392	1380	1380	1378	1382	Stretching vibration of B–O^−^ in BØ_2_O^−^ (meta-borate) units	^50,51^
1464	1482	1462	1464	1462	1462	Stretching vibrations of B–Ø in BØ_3_ units	^50,51^

The observable broadband near ~ 476 cm^−1^ is assigned to (1) cationic vibrations of (Na^+^, Li^+^, Zr^4+^, Fe^3+^, (2) octahedron [ZrO_6_]^2−^ vibrations, (3) vibrations of octahedral units of FeO_6_^44,47,52,53^. A small band ~ 500 cm^−1^ representing the stretching vibration of ZrO_4_ units. The band at ~ 450 cm^−1^ has shifted to ~ 476 cm^−1^ as Fe_2_O_3_ replaced ZrO_2_ in the glass structure. Furthermore, the band at ~ 500 cm^−1^ converges with the band at ~ 476 cm^−1^, resulting in the reduction of ZrO_4_ units in the glass, which replaces FeO_4_ tetrahedral and FeO_6_ octahedral units in the glass network^44,45^. The peak ranging ~ 704 cm^−1^ to ~ 706 cm^−1^ represents bending vibrations of B–O–B linkages in borate two triangular series [O_2_B–O–BO_2_] with Zr–O–Zr linkages in ZrO_4_ units. However, as the concentration of Fe_2_O_3_ increases, the ZrO_4_ units will replace the vibration of Fe–O bonds in FeO_4_ units in the glassy network^34,44,45^. The peak ~ 823 cm^−1^ to ~ 838 cm^−1^ and ~ 863 cm^−1^ to ~ 888 cm^−1^ attributed to B–O–B bending of tri-, tetra- and penta-borate units^45,46^. The more intense bands ~ 926 cm^−1^ to ~ 947 cm^−1^ and ~ 992 cm^−1^ to ~ 1006 cm^−1^ are tailored to diborate units [B_4_O_7_^2−^] of BO_4_ or [BØ_4_]^−^ groups, where Ø denotes bridging oxygen (BO) and O^−^ denotes non-bridging oxygen (NBO_S_) in the glass network^46–48^. The peaks ~ 1065 cm^−1^ to ~ 1075 cm^−1^ are ascribed to stretching vibrations of various (tri-, tetra- and penta-) borate groups in BO_4_ units^49^. The BO_3_ groups create boroxol rings (B_3_O_4,5_)·BO_4_ groups can be integrated into a variety of boroxol groups, forming from di-, tri-, tetra, and penta-borate groups into ortho-, meta-, and pyro-borate groups. The small shoulder bands ~ 1226 cm^−1^ to ~ 1236 cm^−1^ are attributed to the formation of BO_3_^3−^ (ortho-borate) units^46^. The intense peaks ~ 1307 cm^−1^ to ~ 1309 cm^−1^ are imputed to the formation of B_2_O_5_^4−^ (pyro-borate dimers) in the network of the glass^50^. The band ~ 1378 cm^−1^ to 1392 cm^−1^ is ascribed to the stretching vibration of B–O^−^ in BØ_2_O^−^ (meta-borate) units. The smaller shoulder peaks ~ 1462 cm^−1^ to ~ 1482 cm^−1^ represent the asymmetric stretching vibrations of B–Ø in BØ_3_ units^50,51^.

The contraction of the BO_4_ units concerning the broadening of the BO_3_ units is seen in the spectra from BNLZF-00 to BNLZF-10 glasses. Major reduction of the intensity of diborate and pentaborate units from BNLZF-00 to BNLZF-10 with an increase in intensity of ortho-borate (BO_3_^3−^) units, and the shift in peaks is observed in the deconvoluted spectra. This explains the conversion of diborate and pentaborate units into ortho-borate units in the network of the glass as the concentration of the Fe_2_O_3_ is increased in the glass system. To sum up, an extra oxygen from Fe_2_O_3,_ substituting the ZrO_2_, has increased the density and the formation of BO_3_^3−^ leads to the NBOs in the glass network with an open structure. This formation of NBOs was confirmed by calculating the N4 parameter, N4 = (area of BO_4_ groups)/(area of BO_4_ groups + area of BO_3_ groups)^26,54^, which explains the ratio of BO_4_ and BO_3_ groups in the glass network. The calculated N4 parameter values of BNLZF-00 to BNLZF-10 are 0.5837, 0.5709, 0.5630, 0.5464, 0.5206, and 0.4952, respectively. The constant decreases in N4 parameters explain the formation of NBOs in the glassy network, where BO_4_ units are converting into BO_3_ units.

### Raman Spectroscopy

**Fig. 10:**
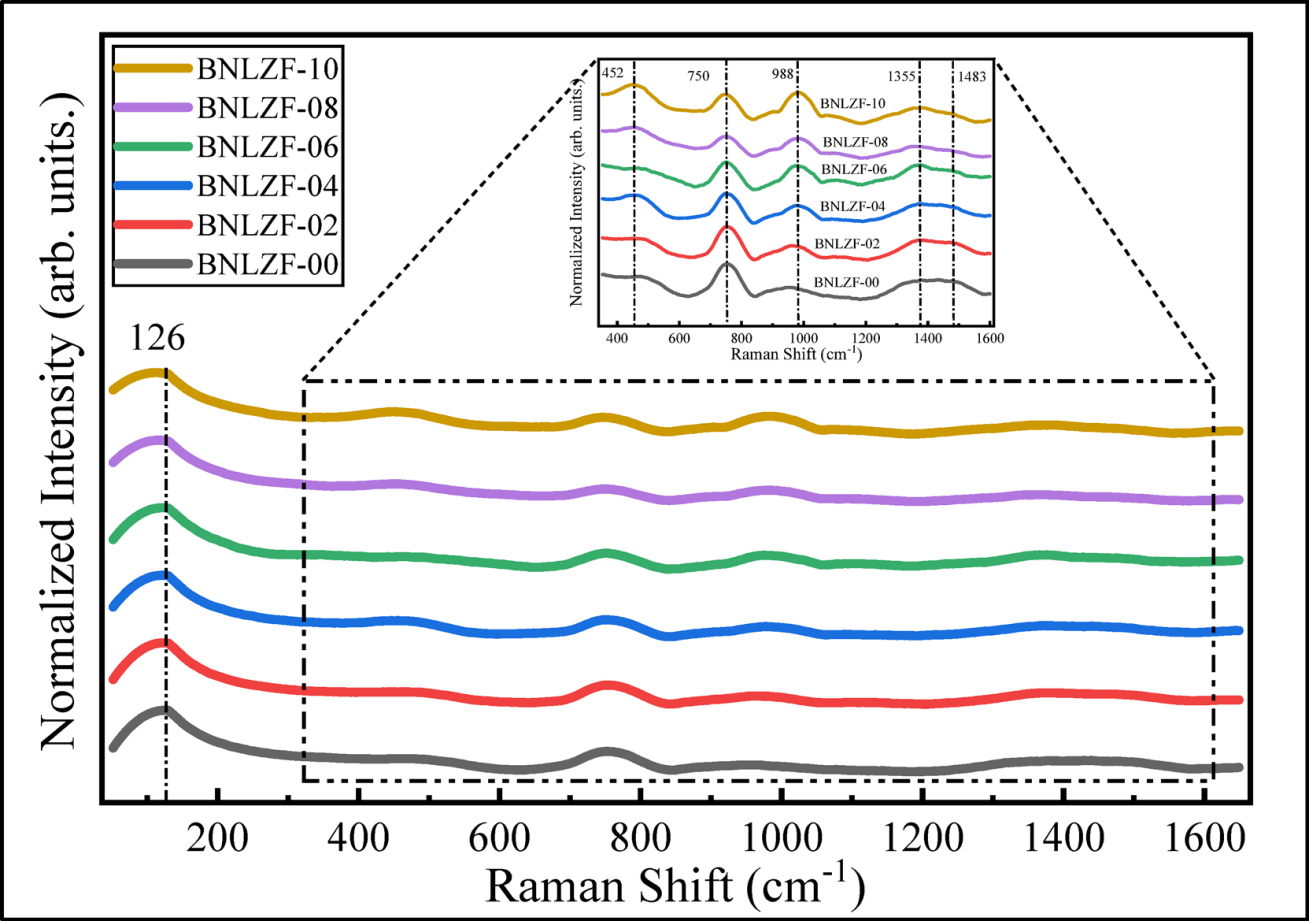
Raman spectra of BNLZF glasses.

**Fig. 11:**
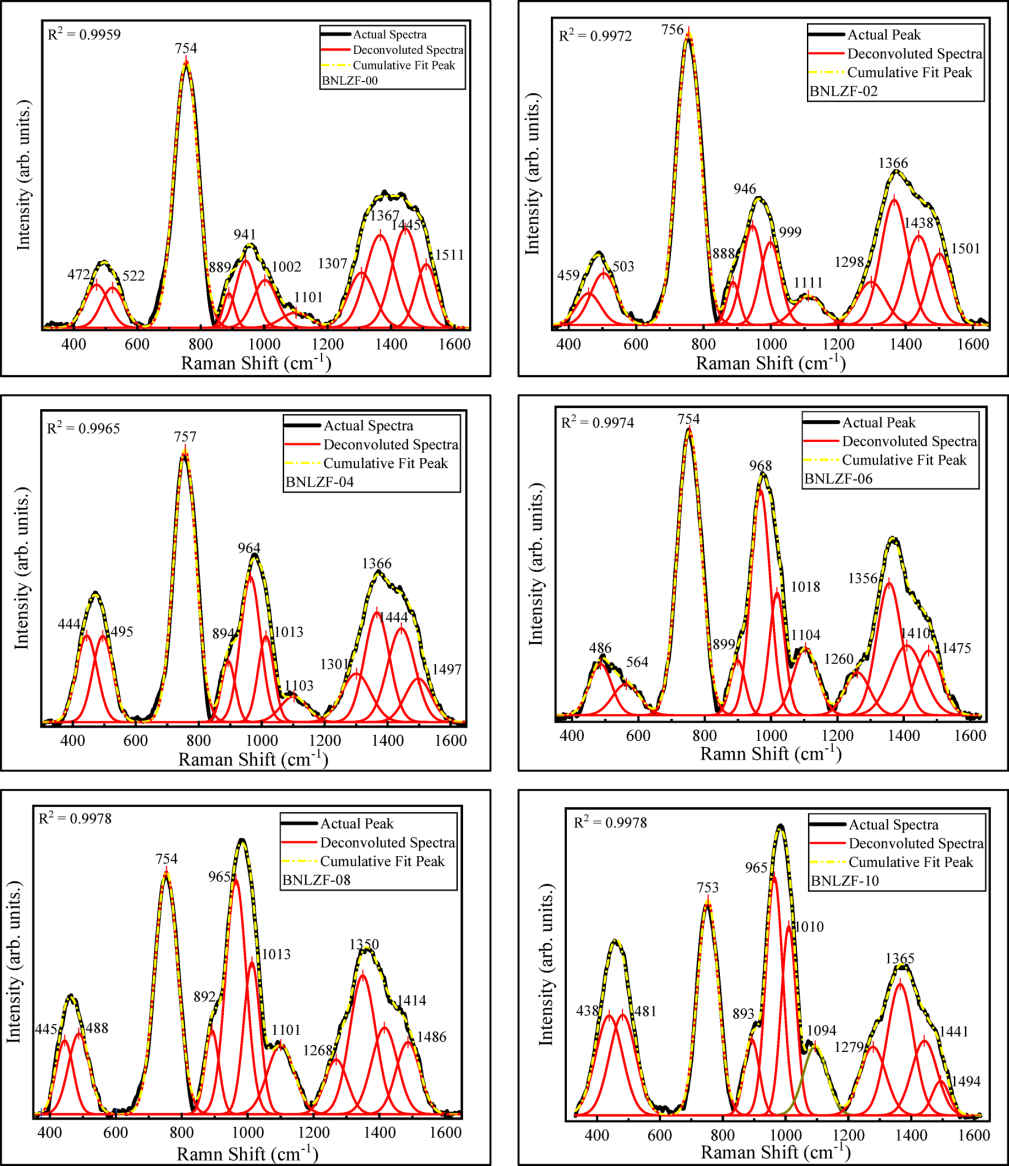
Deconvolution Raman spectra of BNLZF glasses.

Raman spectroscopy is an effective method for examining the structural features of non-crystalline materials. This method thoroughly reveals the local atomic configurations and the impact of different dopants and modifiers on the glass structure. The Raman spectra of the BNLZF glasses were displayed in Fig. 10. The deconvolution was conducted to identify the band assignments and is illustrated in Fig. 11, which is listed in Table 4. The bands have been separated into five sections for each of the synthesized glasses (1) < 200 cm^−1^, (2) 200–400 cm^−1^, (3) 400–800 cm^−1^, (4) 800–1200 cm^−1^, and (5) 1200–1600 cm^−1^^55^.

**Table 4:** Band assignments of deconvoluted Raman spectra of BNLZF glasses.

BNLZF-00	BNLZF-02	BNLZF-04	BNLZF-06	BNLZF-08	BNLZF-10	Band assignments	Refs.
474	459	444	486	445	438	[ZrO_4_] unit’s vibrations in Zr–O linkages	^22,46,56^
522	503	495	564	488	481	Stretching of symmetrical bands of BO_4_ units	
754	756	757	754	754	753	Li-pentaborate and di-Na-pentaborate	^56,57^
889	888	894	899	892	893	Pyroborate units in BO_4_ units	^24,46,58,59^
941	946	964	968	965	965	Orthoborate units with Li/Na–O–Fe vibrations	
1002	999	1013	1018	1013	1010		
1101	1111	1103	1104	1101	1094	Different diborate groups	^46,58^
1307	1298	1301	1260	1268	1279	Pyroborate groups in BO_3_ units	^30,46,57^
1367	1366	1366	1356	1350	1365	BO_4_^−^ linked to BO_2_O^−^ triangles	^30^
1445	1438	1444	1410	1414	1441	B–O^−^ bonds or BO_2_O^−^ triangles (NBOs)	^46,58^
1511	1501	1497	1475	1486	1494		

The highest intensity band, ~ 126 cm^−1^, referred to the boson peak due to the presence of alkali oxides (Li_2_O and Na_2_O) in the glass network^22,55^. The band between ~ 438 and ~ 486 cm^−1^ is attributed to [ZrO_4_] unit’s vibrations in Zr-O linkages, and the bands ~ 481 cm^−1^ to ~ 564 cm^−1^ are assigned to the stretching of symmetrical bands of BO_4_ units, which are surrounded by pairs of BO_3_ units or the isolated diborate groups^22,46,56^. The strong band ~ 753 cm^−1^ to ~ 757 cm^−1^ is imputed to the presence of di-Na-pentaborate and Li-pentaborate due to mixed alkali oxides present in the glassy network^56,57^. The shoulder band ~ 888 cm^−1^ to ~ 899 cm^−1^ is ascribed to the presence of a small amount of pyroborate units in BO_4_ units. The rising band ~ 941 cm^−1^ to ~ 968 cm^−1^ and the shoulder band ~ 999 cm^−1^ to ~ 1018 cm^−1^ is attributed to increased orthoborate units with Li/Na–O–Fe vibrations^24,46,58,59^. The small emerging band ~ 1094 cm^−1^ to ~ 1111 cm^−1^ is imputed to different diborate groups^46,58^. The band ~ 1260 cm^−1^ to ~ 1307 cm^−1^ is assigned to existence of pyroborate groups in BO_3_ units^30,46,57^. The emerging band ~ 1350 cm^−1^ to ~ 1367 cm^−1^ is attributed to BO_4_^−^ linked to BO_2_O^−^ triangles in the glass^30^. The shoulder bands ~ 1410 cm^−1^ to ~ 1445 cm^−1^ and ~ 1475 cm^−1^ to ~ 1511 cm^−1^ is ascribed to extension of B–O^−^ bonds or BO_2_O^−^ triangles (NBOs) connected to an enormous borate group^46,58^.

**Fig. 12:**
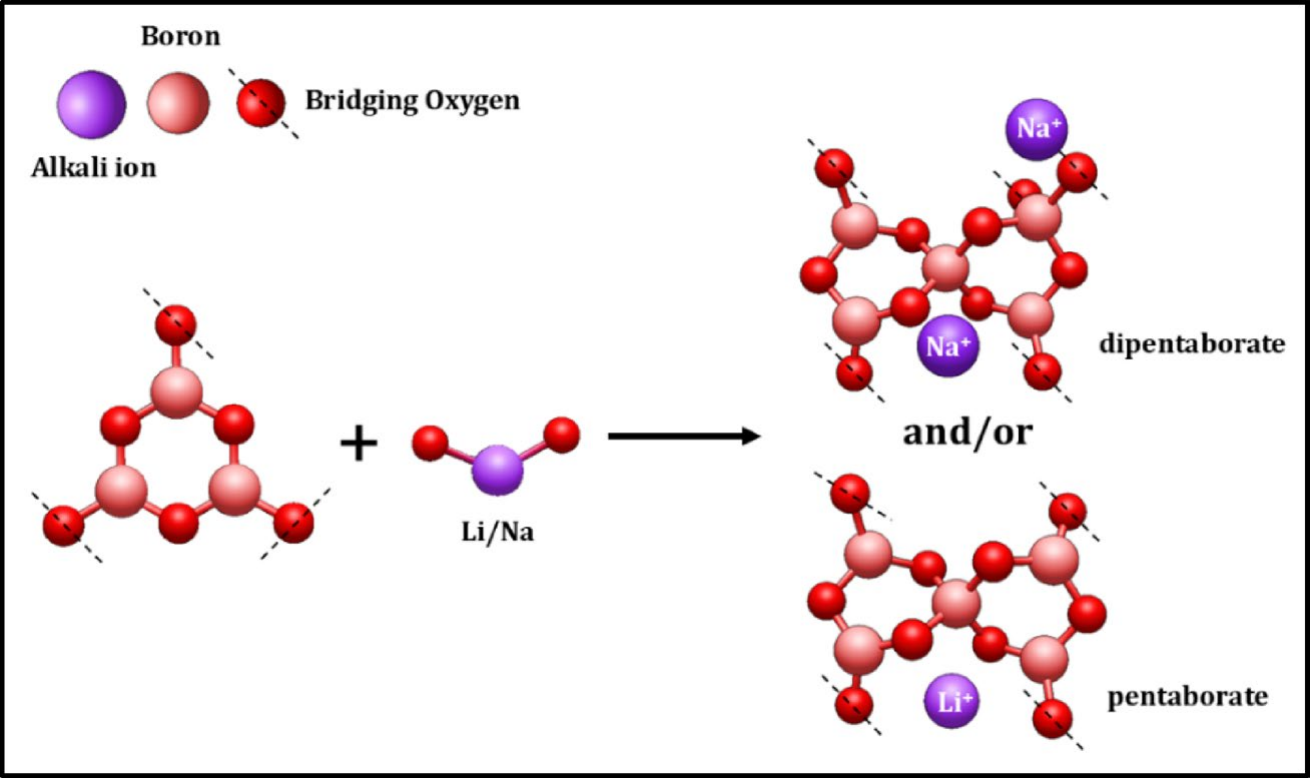
Formation of pentaborate and di-pentaborate in BNLZF-00 glass.

**Fig. 13:**
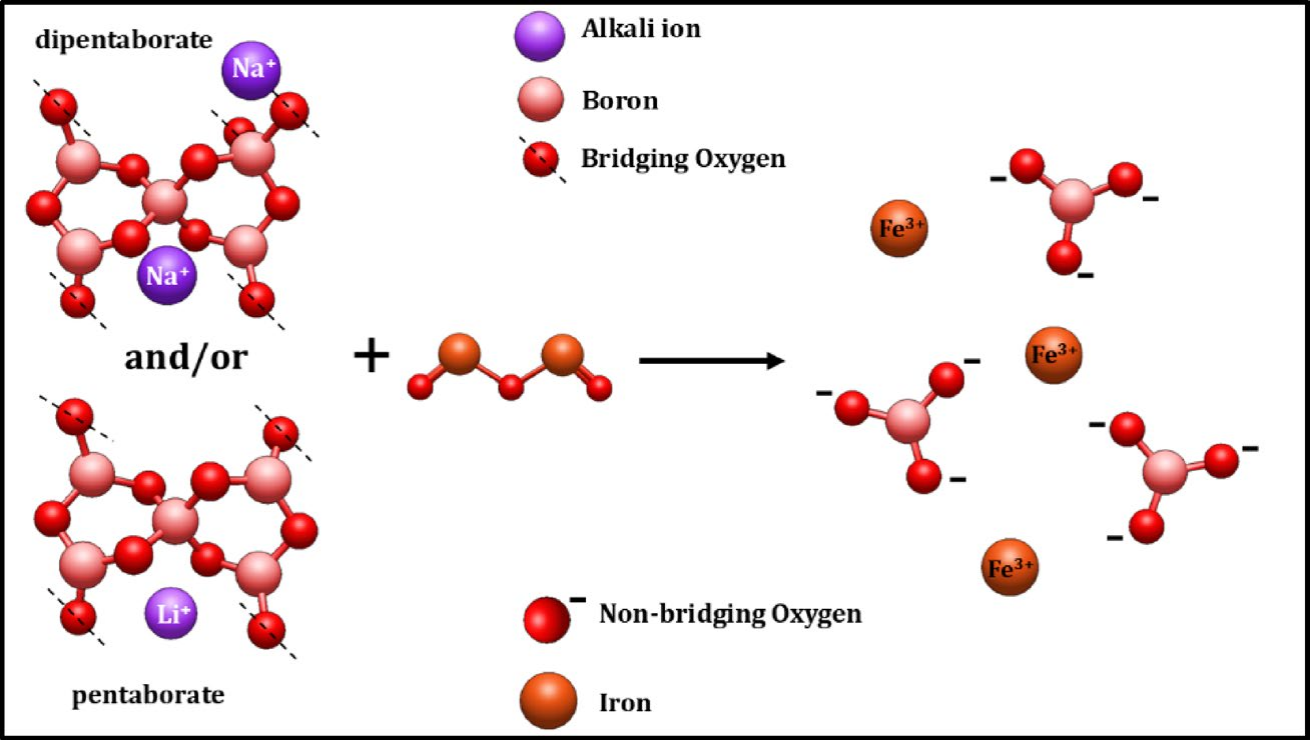
Formation of pentaborate and di-pentaborate to orthoborate in BNLZF-02 to BNLZF-10 glasses.

The presence of mixed-alkali oxides in the glass is higher with respect to the B_2_O_3_. This leads to the formation of pentaborate and di-pentaborate with the Li^+^ and Na^+^ ions, as displayed in Fig. 12. This complex structure will occur only with a high concentration of alkali oxides (Li_2_O and Na_2_O) in the base glass coded BNLZF-00. As the concentration of Fe^3+^ increases, Fe_2_O_3_ partially replaces ZrO_2_ and changes the glass network. The addition of Fe^3+^ ions disrupts the B–O–B bonds, leading to the creation of NBOs. The major formation of the orthoborate units is seen on behalf of pentaborate and di-pentaborate groups, as shown in Fig. 13. Since the formation of orthoborate leads to the formation of NBOs, some stretching vibration of BO_4_ units with vibrations of Zr–O linkages were also observed in the network. This leads to the formation NBOs in the glassy network by the formation of an open structure, evidenced in the FTIR spectra.

### Optical properties

#### Optical absorption

**Fig. 14:**
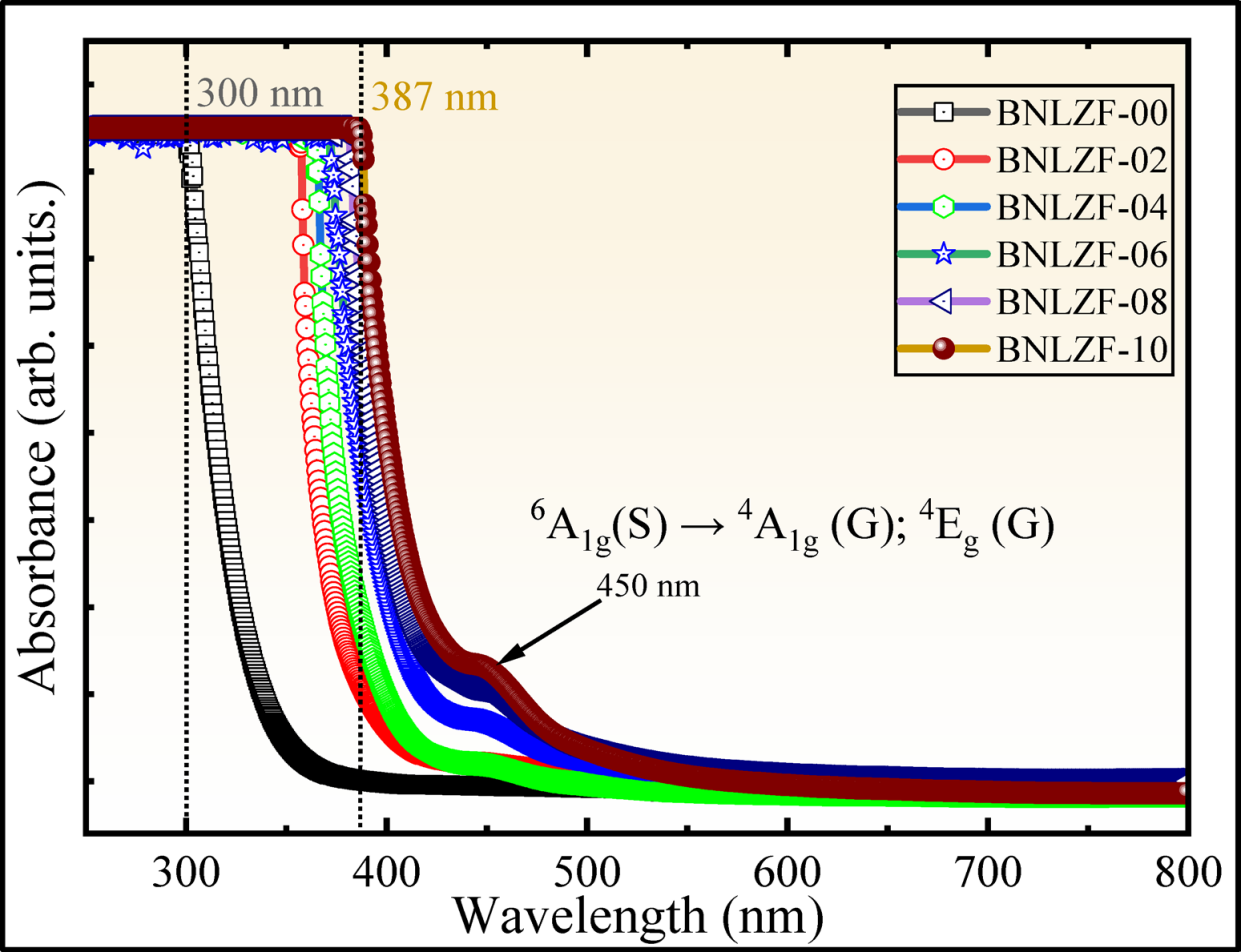
UV–Vis spectra of BNLZF glasses.

Figure 14 illustrates the UV–Vis optical absorption spectra of the present BNLZF glasses. Additionally, it is noted that as Fe_2_O_3_ concentration increases, the absorption edge moves towards the longer wavelength, causing a redshift from 300 to 387 nm. This perceived redshift may be due to the transition of the electrons to the excited state from the valence band of the oxygen atom^60^. Among all the TMIs in the periodic table, Fe^3+^-ions, along with a d^5^ configuration, are much scrutinised because they mostly exhibit weak prohibited transitions rather than a spin-allowed transition. The UV–Vis absorption band of the Fe^3+^ ions is observed to increase at ~ 450 nm with an increase in Fe_2_O_3_ concentration. This band is ascribed to ^6^A_1g_ (^6^S) → ^4^A_1g_ (^4^G); ^4^E_g_ (^4^G), a *d*–*d* transition of Fe^3+^ ions in the tetrahedral and/or distorted octahedral symmetrical structure in the network of the BNLZF glass^24,61^. The interaction of photons increases as the Fe^3+^ ion concentration is more in the glass matrix, with the reduction in the optical bandgap.

#### Optical bandgap (${{\boldsymbol{E}}}_{{\boldsymbol{o}}{\boldsymbol{p}}{\boldsymbol{t}}}$)

**Fig. 15:**
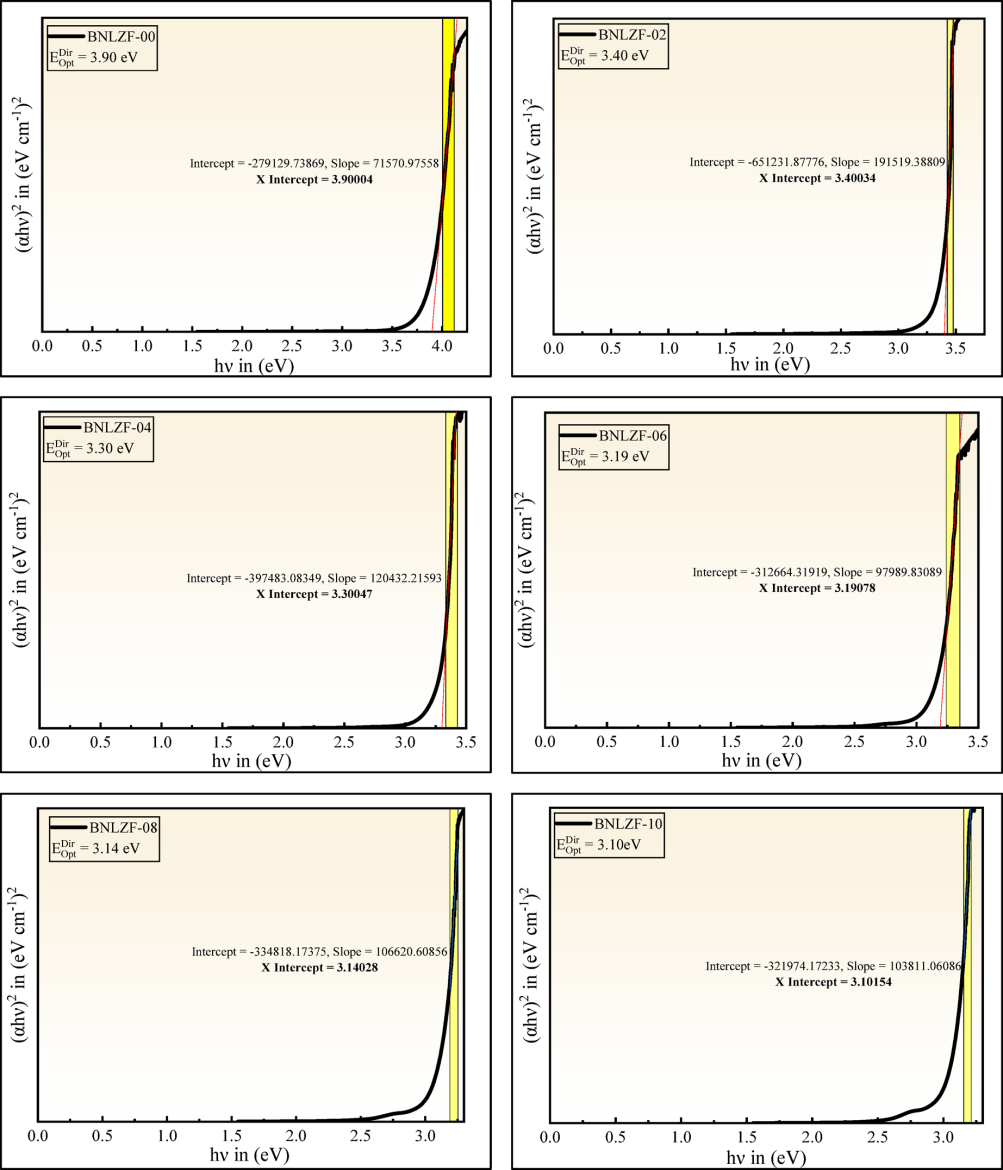
Direct bandgap of BNLZF glasses.

**Fig. 16:**
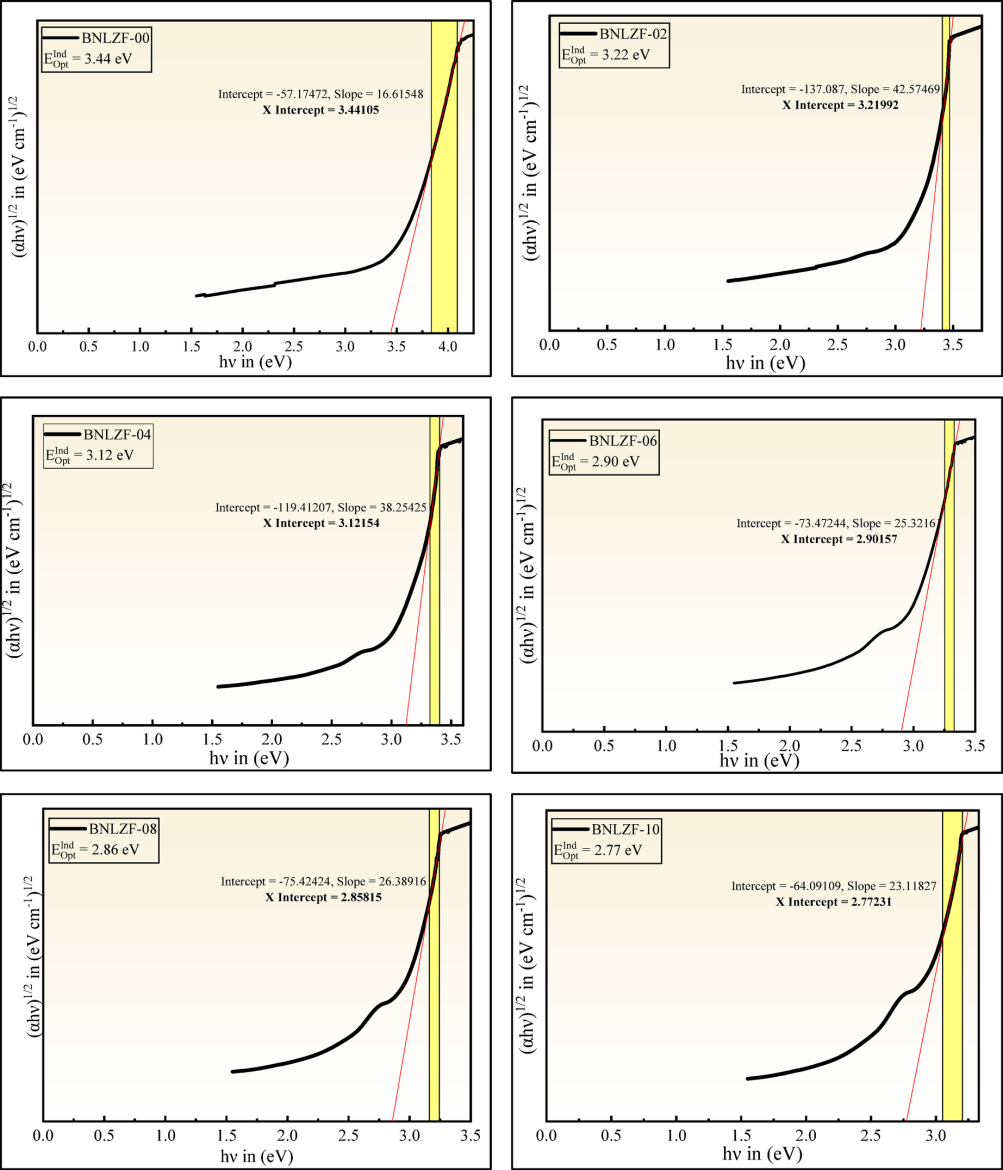
Indirect bandgap of BNLZF glasses.

Optical parameters are extracted from the optical absorption edge in the Tauc region (high-energy region) by absorption spectra. The Beer-Lambert law applies in amorphous glass systems because the samples are optically uniform and free of scattering centres, allowing absorbance to be immediately transformed into the absorption coefficient. Therefore, the optical absorption coefficient, $\alpha (\nu )$ is evaluated by the following Eq. (11).

11 $$\alpha \left(\nu \right)=2.303 \left(A/d\right)$$

where $A$ and $d$ are the absorbance in UV–Vis spectra and the glass sample thickness, respectively. This relationship is suitable for the current glass samples science light attenuation primarily results from intrinsic electronic absorption close to the band edge, ensuring accurate $\alpha (\nu )$ estimation for Tauc analysis. Further, the ${E}_{opt}$ is calculated by the obtained optical absorption coefficient using Davis–Mott’s expression given in Eq. (12).

12 $$\alpha h\nu =T{(h\nu -{E}_{opt})}^{i}$$

where $h$ is Planck’s constant, $h\nu$ is the incident photon energy, T is the band tailing parameter. The values of the index $i$ are chosen according to the selected electronic transition: direct allowed, direct forbidden, indirect allowed, and indirect forbidden, with the values 1/2, 3/2, 2, and 3, respectively^62,63^. The linear section of the obtained curve is extended as ${(\alpha h\nu )}^{2}$ tends to 0 and ${(\alpha h\nu )}^{1/2}$ tends to 0 on the x-axis in the plotted graph of direct bandgap and indirect bandgap values that are listed in Table 5. The direct bandgap $\left({E}_{Opt}^{Dir}\right)$ reduced from 3.90 to 3.10 eV, and the indirect bandgap $\left({E}_{Opt}^{Ind}\right)$ reduced from 3.44 to 2.77 eV, displayed in Figs. 15 and 16, respectively. As a result, optical bandgap decreases, proving overall bandgap values are enhanced compared to those reported in previous investigations were listed in Table 6. The creation of NBOs due to an increase in the concentration of Fe_2_O_3_ leads to the reduction of the optical bandgap.

**Table 5:** Optical parameters of the BNLZF glasses with error range ± 0.001.

Sample code	BNLZF-00	BNLZF-02	BNLZF-04	BNLZF-06	BNLZF-08	BNLZF-10
${E}_{Opt}^{Dir}$ (eV)	3.90	3.40	3.30	3.19	3.14	3.10
${E}_{Opt}^{Ind}$ (eV)	3.44	3.22	3.12	2.90	2.86	2.77
${U}_{E}$ (eV)	0.245	0.247	0.251	0.263	0.270	0.273
${\sigma }_{S}$	0.106	0.105	0.103	0.098	0.096	0.095
${E}_{(e-ph)}$	6.302	6.359	6.466	6.768	6.945	7.039
$n$	1.54	1.55	1.55	1.56	1.56	1.57
$\varepsilon$	2.372	2.387	2.403	2.418	2.434	2.465
${\varepsilon }_{opt}$	1.372	1.387	1.403	1.418	1.434	1.465
${\chi }_{e}$	0.109	0.110	0.112	0.113	0.114	0.117
${R}_{L}$	4.52	4.59	4.65	4.72	4.79	4.92
${T}_{C}$	91.35	91.23	91.11	90.99	90.87	90.62
$NA$	0.2178	0.2185	0.2192	0.2199	0.2206	0.2220
${R}_{m}$ (cm^3^)	15.01	15.25	15.45	15.65	15.85	16.16
${\alpha }_{m}$ (Å^3^)	5.96	6.05	6.13	6.21	6.29	6.41
$M$	0.686	0.684	0.681	0.679	0.677	0.672
$\chi$	0.925	0.866	0.839	0.780	0.769	0.745
${\alpha }_{0}$	2.668	2.721	2.745	2.798	2.808	2.830
${\alpha }_{{O}^{2-}}(n)$ (Å^3^)	2.538	2.577	2.608	2.640	2.672	2.722
${\alpha }_{{O}^{2-}}\left({E}_{Opt}^{Ind}\right)$ (Å^3^)	4.780	4.927	5.002	5.144	5.189	5.258
${\Lambda }_{\chi }$	1.238	1.267	1.281	1.310	1.316	1.328
${\Lambda }_{n}$	1.012	1.022	1.030	1.038	1.045	1.056
${\Lambda }_{{E}_{Opt}^{Ind}}$	1.321	1.331	1.336	1.345	1.348	1.352
${n}_{2}$ × 10^−13^ (esu)	5.90	6.15	6.41	6.68	6.96	7.54
${\chi }^{(3)}$ × 10^−14^ (esu)	2.41	2.52	2.64	2.75	2.88	3.14

**Table 6:** Compared optical parameters with BNLZF glass.

Glass matrix	Optical band gap, eV	Electronic oxide ion polarizability, Å^3^	Optical basicity	Third-order susceptibility, × 10^−14^ (esu)	Non-linear refractive index, × 10^−13^ (esu)	Refs.
60B_2_O_3_–25Na_2_O–10Li_2_O–4ZrO_2_–1Fe_2_O_3_	2.77	5.258	1.352	3.14	7.54	Present Work
35Bi_2_O_3_–40B_2_O_3_–24TeO_2_–1Fe_2_O_3_	2.7601	3.5807	1.2036			^64^
35P_2_O_5_–40ZnO–25Na_2_O–1Fe_2_O_3_	2.869	–	–	–	–	^65^
59P_2_O_5_–37PbO_2_–3As_2_O_3_–1Fe_2_O_3_	2.78	–	0.764	0.3327	0.5043	^66^
65B_2_O_3_–15NaF–15ZnO–5Bi_2_O_3_–0.5Fe_2_O_3_–0CuO	3.595	–	–	–	–	^67^
10SiO_2_–65B_2_O_3_–24Na_2_O–1Fe_2_O_3_	2.97	–	1.04	3.58	5.61	^68^

### Urbach energy (${{\boldsymbol{U}}}_{{\boldsymbol{E}}}$) and steepness parameter (${{\boldsymbol{\sigma}}}_{{\boldsymbol{S}}}$)

The localized bandgap defect state, which blocks the electrons from transitioning directly from the valence band to the conduction band, is known as the Urbach energy. These localized bandgap defects cause the tail that is frequently seen in the optical absorption spectra and extends into the forbidden bandgap. The Urbach energy (${U}_{E}$) is measured by the width of the tail, which is related to the distortedness in the localized bandgaps. The following Eq. (13) determines the ${U}_{E}$^63^.

13 $$\alpha ={\alpha }_{0}{e}^{h\nu /{U}_{E}}$$

**Fig. 17:**
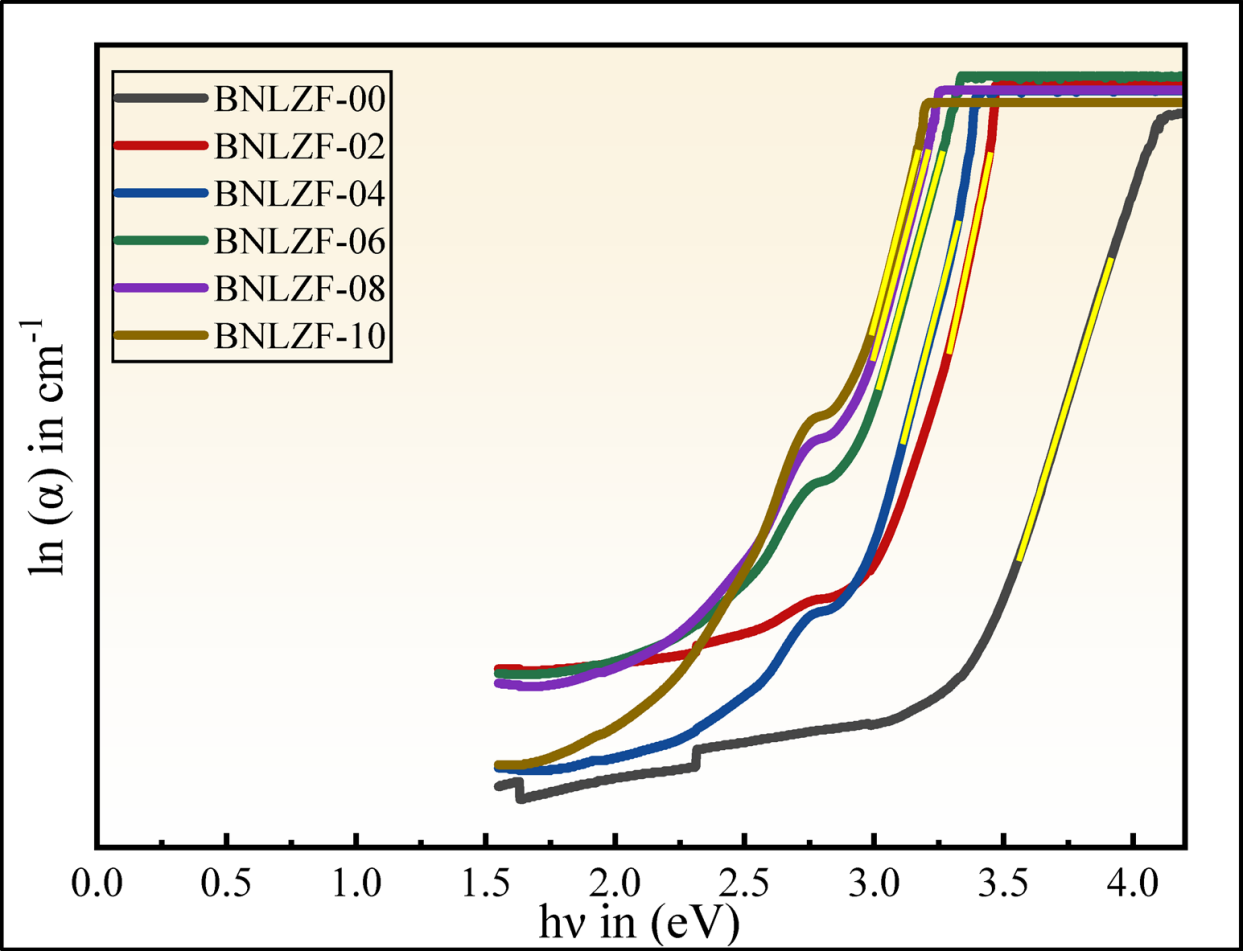
The Urbach energy of BNLZF glasses.

The graph $\mathrm{ln}(\alpha )$ vs. $h\nu$ (photon energy) is drawn to obtain the ${U}_{E}$, where ${\alpha }_{0}$ is a constant. The ${U}_{E}$ is calculated by obtaining the inverse of the slope as determined by Fig. 17^69^.

14 $${U}_{E}=\frac{1}{slope}$$

**Fig. 18:**
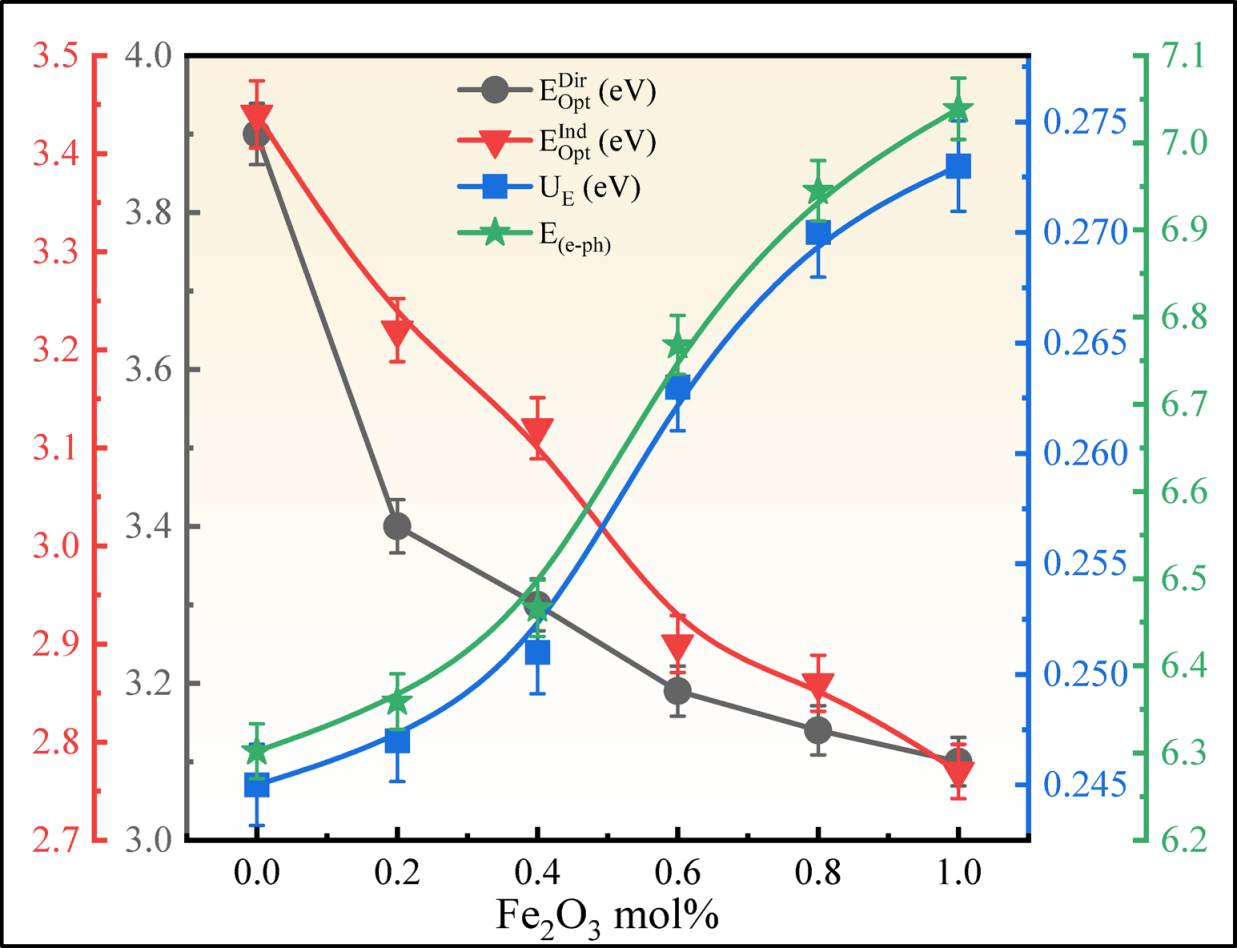
Comparison between ${E}_{Opt}^{Dir}$, ${E}_{Opt}^{Ind}$, ${U}_{E}$, and ${E}_{(e-ph)}$ of BNLZF glasses.

The calculated ${U}_{E}$ values are tabulated in Table 5. The values of ${U}_{E}$ increased from 0.245 to 0.273 eV as the concentration of Fe_2_O_3_ is replaced with ZrO_2_, compared with the bandgap in Fig. 18. An increase in Fe^3+^ concentration creates localised states and NBOs in the glass network, leading to structural disorder. As a result, the optical bandgap decreases but the Urbach energy increases, indicating the degree of disorder in the glass matrix. The highest degree of disorderedness of obtained in BNLZF-10 glass. This explains the increase in disorderedness by Fe_2_O_3_, creating the open structure in the network of the glass by the formation of NBOs explained in FTIR and Raman spectroscopy.

The interactions of exciton-phonon and/or electron–phonon (${E}_{(e-ph)}$) broaden the absorption edge, which is estimated from the steepness parameter ${\sigma }_{S}$, which is calculated by the following Eqs. (15) and (16)^69,70^.

15 $${\sigma }_{S}={k}_{B}T/{U}_{E}$$

16 $${E}_{(e-ph)}=2/3{\sigma }_{S}$$

where ${k}_{B}$ and $T$ are the Boltzmann constant and the room temperature (300 K), respectively. The computed data are listed in Table 5, and the decrease in values ${\sigma }_{S}$ from 0.106 to 0.095, and the gradual increase in ${E}_{(e-ph)}$ from 6.302 to 7.039 due to the ionicity effect in glass and distinct anion charges in the current BNLZF glass, as shown in Fig. 18^70^.

### Dielectric constant (${\boldsymbol{\varepsilon}}$), optical dielectric constant (${{\boldsymbol{\varepsilon}}}_{{\boldsymbol{o}}{\boldsymbol{p}}{\boldsymbol{t}}}$), and linear dielectric susceptibility (${{\boldsymbol{\chi}}}_{{\boldsymbol{e}}}$)

The dipole orientation and refractive index contribute towards the polarizability of the glass, which contains a strong relationship with the values of $\varepsilon$, ${\varepsilon }_{opt}$, and ${\chi }_{e}$, which is shown in the following relations:

17 $$\varepsilon ={n}^{2}$$

18 $${\varepsilon }_{opt}={n}^{2}-1$$

19 $${\chi }_{e}=\frac{{n}^{2}-1}{4\pi }=\frac{{\varepsilon }_{opt}}{4\pi }$$

The calculated values are tabulated in Table 5. The measured values of the refractive index ($n$) varied from 1.54 to 1.57 and tabulated in Table 5. The $\varepsilon$ values increased from 2.372 to 2.465, ${\varepsilon }_{opt}$ values increased from 1.372 to 1.465, and ${\chi }_{e}$ values increased from 0.109 to 0.117, which confirms the linear dependency towards the values of $n$^54,69^.

### Reflection loss (${{\boldsymbol{R}}}_{{\boldsymbol{L}}}$), transmittance coefficient (${{\boldsymbol{T}}}_{{\boldsymbol{C}}}$) and numerical aperture (${\boldsymbol{N}}{\boldsymbol{A}}$).

**Fig. 19:**
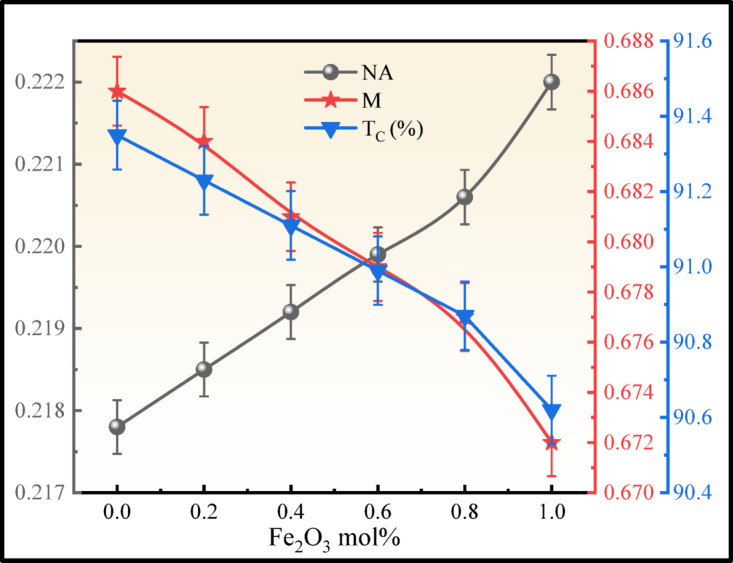
Comparison of $NA$, $M$, and ${T}_{C}$ of BNLZF glass samples.

The following equations are utilized to calculate the reflection loss (${R}_{L}$), transmittance coefficient (${T}_{C}$), and numerical aperture ($NA$).

20 $${R}_{L}={\left(\frac{n-1}{n+1}\right)}^{2}\times 100\%$$

21 $${T}_{C}=\frac{2n}{{n}^{2}+1}\times 100\%$$

22 $$NA{=n\left[2\Delta \right]}^{1/2}$$

where $n$ is the refractive index and the $\Delta$ is the small change in RI (0.01). The ${R}_{L}$ increased from 4.52 to 4.92% and ${T}_{C}$ decreased from 91.35 to 90.62% due to adding Fe_2_O_3_ into the glass network. The value of $NA$ ranging from 0.13 to 0.5 will be used as the core material in glass optical fibre cable. The $NA$ of the present BNLZF glasses vary from 0.2178 to 0.222, and are compared in Fig. 19, which is considered a promising material for the core part of optical fiber cables^42,54,69,70^. The values are displayed in Table 5.

### Molar refraction (${{\boldsymbol{R}}}_{{\boldsymbol{m}}}$), molar electronic polarizability (${\boldsymbol{\alpha }}_{{\boldsymbol{m}}}$), and Metallization criterion (${\boldsymbol{M}}$)

The following Eqs. (23), (24), and (25) are obtained to compute the ${R}_{m}$, ${\alpha }_{m}$, $M$ to understand the molar polarizability and metallic nature of the BNLZF glass. The refractive index of the glass and the Lorentz-Lorentz equation are utilized to determine the metallic/non-metallic nature of the glasses based on ${R}_{m}$:${V}_{m}$ ratio as described by Dimitrov and Komatsu^71^. If ${R}_{m}$:${V}_{m}$ < 1, the material is non-metallic, and if ${R}_{m}$:${V}_{m}$ > 1, the material is metallic. Metallization criterion of the BNLZF glasses is calculated in accordance with the Herzfeld theory of metallization^38,42,63,69,70^.

23 $${R}_{m}=\left[\frac{{n}^{2}-1}{{n}^{2}+2}\right]{V}_{m}$$

24 $${\alpha }_{m}=\frac{{R}_{m}}{2.52}$$

25 $$M=\frac{{R}_{m}}{{V}_{m}}$$

where $n$ is the refractive index and ${V}_{m}$ is the molar volume of the BNLZF glass, and calculated values are tabulated in Table 5, and compared in Fig. 19. ${R}_{m}$ increased to 16.16 cm^3^ from 15.01 cm^3^ and ${\alpha }_{m}$ increased to 6.41 Å^3^ from 5.96 Å^3^ as the Fe^3+^ ions were introduced into the glass network. Whereas $M$ values reduced to 0.672 from 0.686. Additionally, materials with a high cap M value, close to 1, are considered insulators, and those with a low cap M value, close to 0, are considered metals^54,69^. In BNLZF glass influence of Fe^3+^ on the glass leads to enhancing the metallic nature of the glass.

### Electronegativity (${\boldsymbol{\chi}}$), electron polarizability (${\boldsymbol{\alpha }}_{0}$)

The following Eq. (26) is used to calculate electronegativity ($\chi$) which has the ability of an atom to attract the shared electrons to be involved in a chemical bond in the network of the glass, and Eq. (27) is used to calculate electron polarizability (${\alpha }_{0}$) which describes the effect of the external electric field to distort the electron clouds present in the glass structure^70,72^.

26 $${\chi =0.2688 E}_{Opt}^{Ind}$$

27 $${\alpha }_{0}=-0.9\left(\chi \right)+3.5$$

$\chi$ show a reduction in the values from 0.925 to 0.745. A rise in the ${\alpha }_{0}$ values from 2.668 to 2.830 are seen in the BNLZF glasses. These obtained values explain the ionic nature and formation of NBOs in the BNLZF glasses due to the increase in Fe^3+^ concentration. The computed data were listed in Table 5.

### Electronic oxide ion polarizability (${\boldsymbol{\alpha }}_{{{\boldsymbol{O}}}^{2-}}$)

**Fig. 20:**
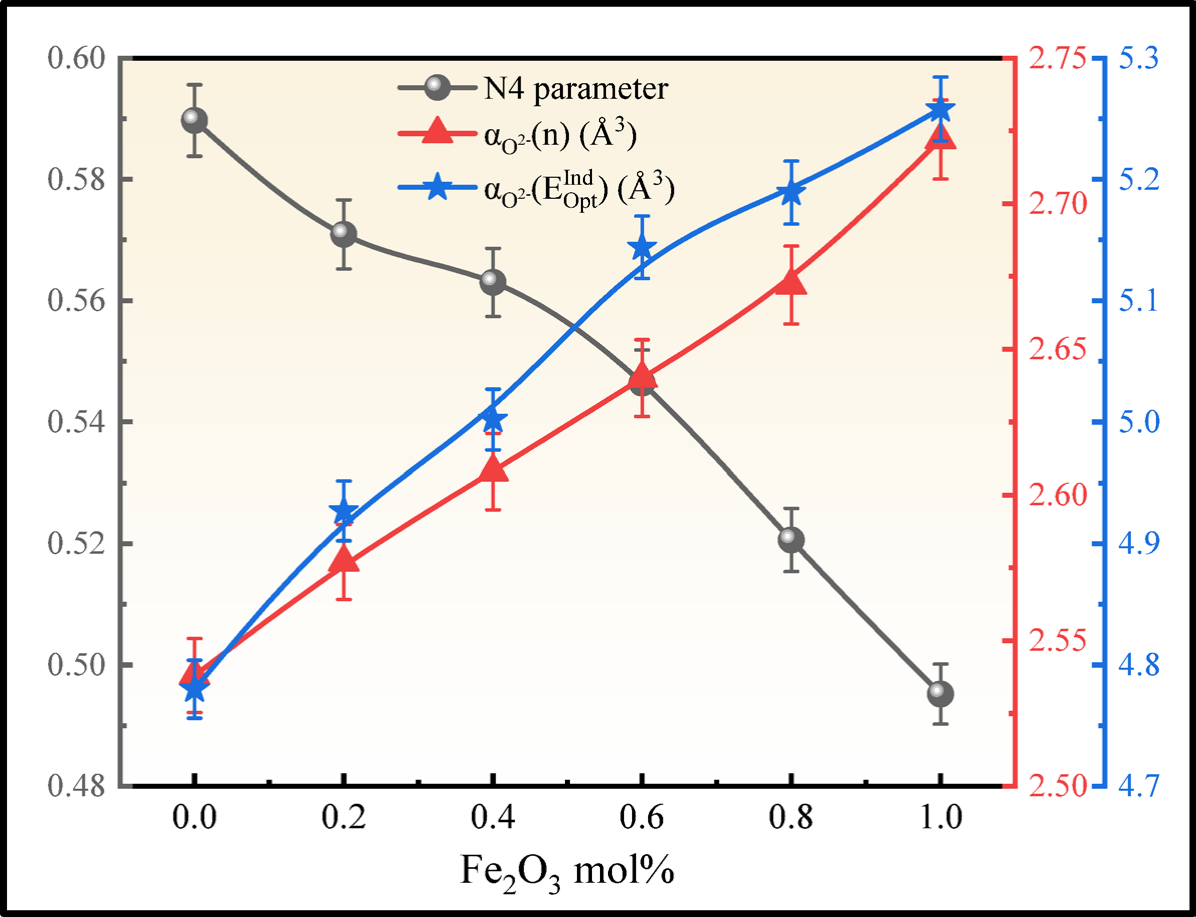
Comparison of N4, $\left({\alpha }_{{O}^{2-}}(n)\right)$, and $\left({\alpha }_{{O}^{2-}}({E}_{Opt}^{Ind})\right)$ of BNLZF glass samples.

The electronic oxide polarizability (${\alpha }_{{O}^{2-}}$) measures the distortion of the electron clouds of oxide ions (O^2−^), which is affected by the external electric field. The equation given below is used to determine ${\alpha }_{{O}^{2-}}$ by refractive index ($n$) and optical bandgap (${E}_{Opt}^{Ind}$)^38,42,54,69^.

28 $${\alpha }_{{O}^{2-}}(n)=\left[\left(\frac{{R}_{m}}{2.52}\right)-\sum {\alpha }_{c}\right]/\left[{N}^{{O}^{2-}}\right]$$

29 $${\alpha }_{{O}^{2-}}\left({E}_{Opt}^{Ind}\right)=\left[\left(\frac{{V}_{m}}{2.52}\right)\left(1-\sqrt{\frac{{E}_{Opt}^{Ind}}{20}}\right)-\sum {\alpha }_{c}\right]/\left[{N}^{{O}^{2-}}\right]$$

where $\sum {\alpha }_{c}$ is molar cation polarizability, which is given by v × 2×${\alpha }_{{B}^{3+}}$ + w × 2×${\alpha }_{{Na}^{+}}$ + x × 2×${\alpha }_{{Li}^{+}}$ + y × 1×${\alpha }_{{Zr}^{4+}}$ + z × 2×${\alpha }_{{Fe}^{3+}}$ from the stoichiometry ratio vB_2_O_3_–wNa_2_O–xLi_2_O–yZrO_2_–zFe_2_O_3_. The values of ${\alpha }_{{B}^{3+}}$ = 0.002 Å^3^, ${\alpha }_{{Na}^{+}}$ = 0.181 Å^3^, ${\alpha }_{{Li}^{+}}$ = 0.029 Å^3^, ${\alpha }_{{Zr}^{4+}}$ = 0.377 Å^3^, ${\alpha }_{{Fe}^{3+}}$ = 0.437 Å^3^ and ${N}^{{O}^{2-}}$ is the number of oxide ions in the glass matrix v × 3 + w × 1 + x × 1 + y × 2 + z × 3. The calculated electronic oxide polarizability using the optical bandgap $\left({\alpha }_{{O}^{2-}}({E}_{Opt}^{Ind})\right)$ and refractive index $\left({\alpha }_{{O}^{2-}}(n)\right)$ is increased from 4.780 to 5.258^64,73^, and from 2.538 to 2.722, respectively. These obtained values are noted in Table 5, and enhanced values are displayed in Table 6. It shows an increasing trend with refractive index and optical bandgap, which is attributed to the formation of NBOs, creating an open structure allowing more distortion in the electron clouds of the oxide ions in the glass network. The comparison of the values is illustrated in Fig. 20.

### Optical basicity ($\boldsymbol{\Lambda }$)

Optical basicity ($\Lambda$) is the quantitative measurement of oxide (II) atoms in electron-donor strength in oxidic networks. The following Eqs. (30), (31), and (32) are used to calculate the $\Lambda$ by electronegativity ($\chi$), electronic ion polarizability with refractive index ($n$), and optical bandgap (${E}_{Opt}^{Ind}$) respectively^42,63,70^.

30 $${\Lambda }_{\chi }=-0.5\chi +1.7$$

31 $${\Lambda }_{n}=1.67\left(1-\frac{1}{{\alpha }_{{O}^{2-}}(n)}\right)$$

32 $${\Lambda }_{{E}_{Opt}^{Ind}}=1.67\left(1-\frac{1}{{\alpha }_{{O}^{2-}}({E}_{Opt}^{Ind})}\right)$$

**Fig. 21:**
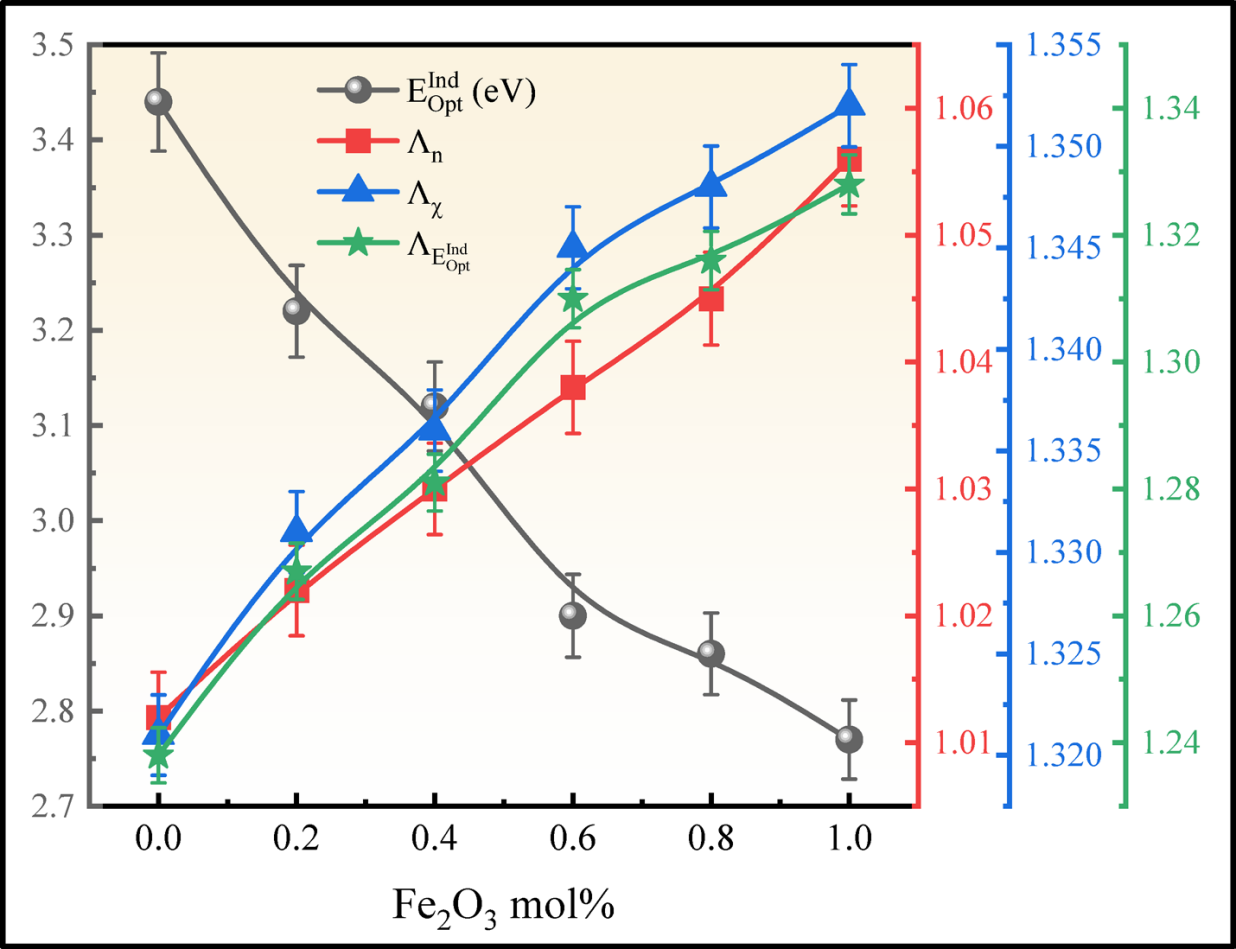
Comparison of optical basicity with ${E}_{Opt}^{Ind}$ of BNLZF glasses.

The optical basicity of the BNLZF glasses was calculated and exemplified in Table 5. ${\Lambda }_{\chi }$, ${\Lambda }_{{E}_{Opt}^{Ind}}$, and ${\Lambda }_{n}$ values are increased from 1.238 to 1.328, from 1.321 to 1.352^64,73^, and from 1.012 to 1.056 as the Fe_2_O_3_ is replaced with ZrO_2_ and the compared values are illustrated in Table 6. The extra oxygen atom from Fe_2_O_3_ replacing ZrO_2_ enhances the optical basicity in the glass system as the oxygen concentration increases in the glass network^74^. The comparison of these values with ${E}_{Opt}^{Ind}$ is displayed in Fig. 21.

### Third-order susceptibility (${{\boldsymbol{\chi}}}^{(3)}$), and non-linear refractive index (${{\boldsymbol{n}}}_{2}$)

**Fig. 22:**
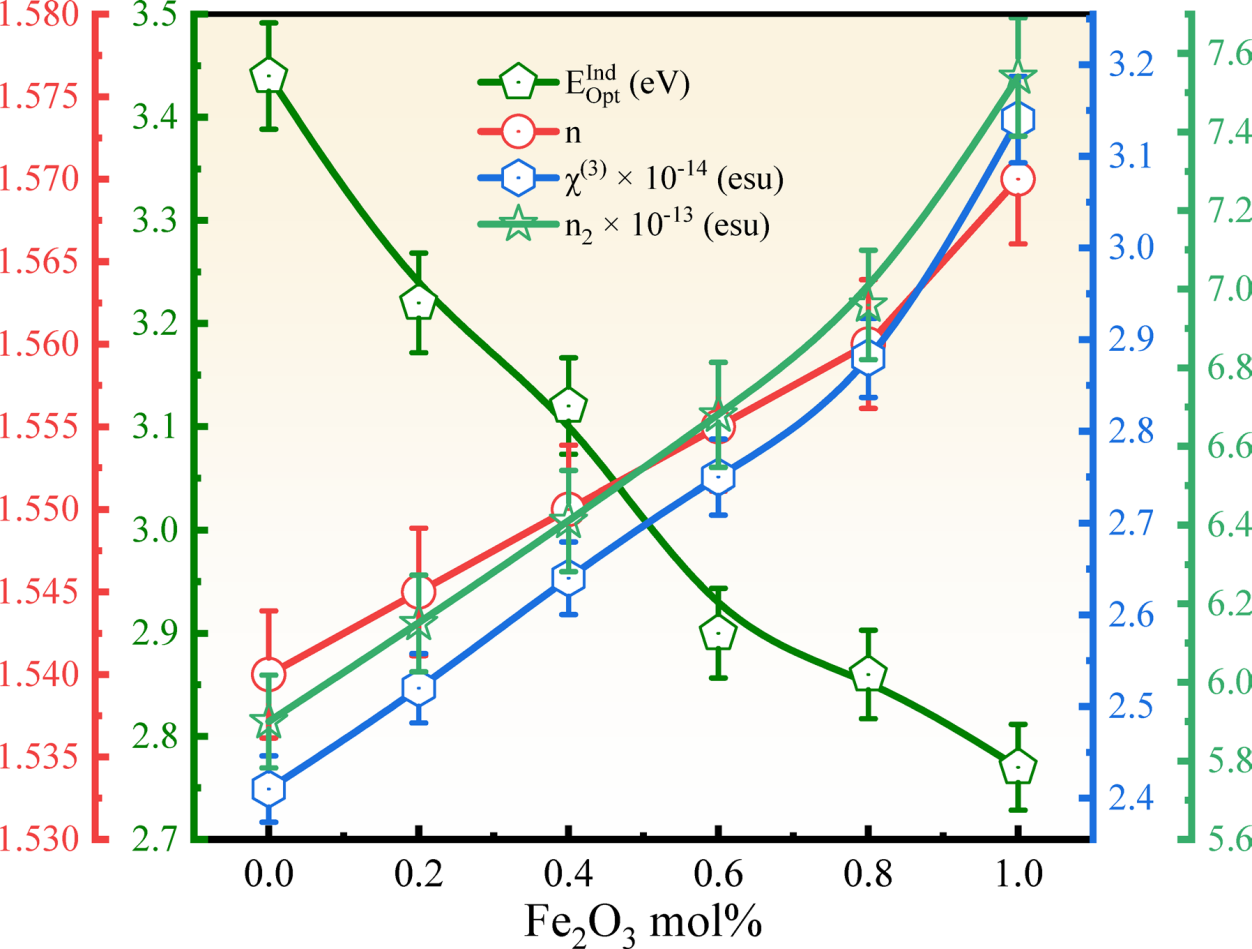
Comparison of ${E}_{Opt}^{Ind}$ with $n$, ${n}_{2}$ and ${\chi }^{(3)}$ of BNLZF glasses.

Third-order susceptibility is directly related to the nonlinear optical (NLO) refractive index, which is the intensity-dependent change in the refractive index of materials. When high-intensity electromagnetic waves interact with dielectric materials, their nonlinear characteristics emerge, making third-order behaviour significant. ${\chi }^{(3)}$ and ${n}_{2}$ Using Ticha-Tichy Eqs. (33) and (34), given below^75^:

33 $${\chi }^{(3)}=A{\left[\frac{{n}^{2}-1}{4\pi }\right]}^{4}$$

34 $${n}_{2}=\frac{12\pi {\chi }^{(3)}}{n}$$

where A (1.7 × 10^−10^ esu) is the constant. The computed data are tabulated in Table 5. The addition of Fe_2_O_3_ to the composition has shown increasing ${n}_{2}$ values increase from 5.90 × 10^−13^ to 7.54 × 10^−13^ esu and ${\chi }^{(3)}$ values increase from 2.41 × 10^−14^ to 3.14 × 10^−14^ esu^76^. These ${n}_{2}$ and ${\chi }^{(3)}$ values are enhanced compared to recent literature in Table 6. Linear and nonlinear susceptibility and refractive index show an increasing trend and are displayed in Fig. 22. As the concentration of Fe^3+^ increases, the refractive index of the glass increases due to increased electronic polarizability and localised electronic states. An increase in the refractive index values promotes the interaction between the electromagnetic field and the glass network, leading to an increase in the ${n}_{2}$ and ${\chi }^{(3)}$ values. This makes the current BNLZF glasses suitable for a wide range of applications in nonlinear optical instruments, optical switching, and advanced communication technology^77^.

### Photoluminescence (PL) spectra

**Fig. 23:**
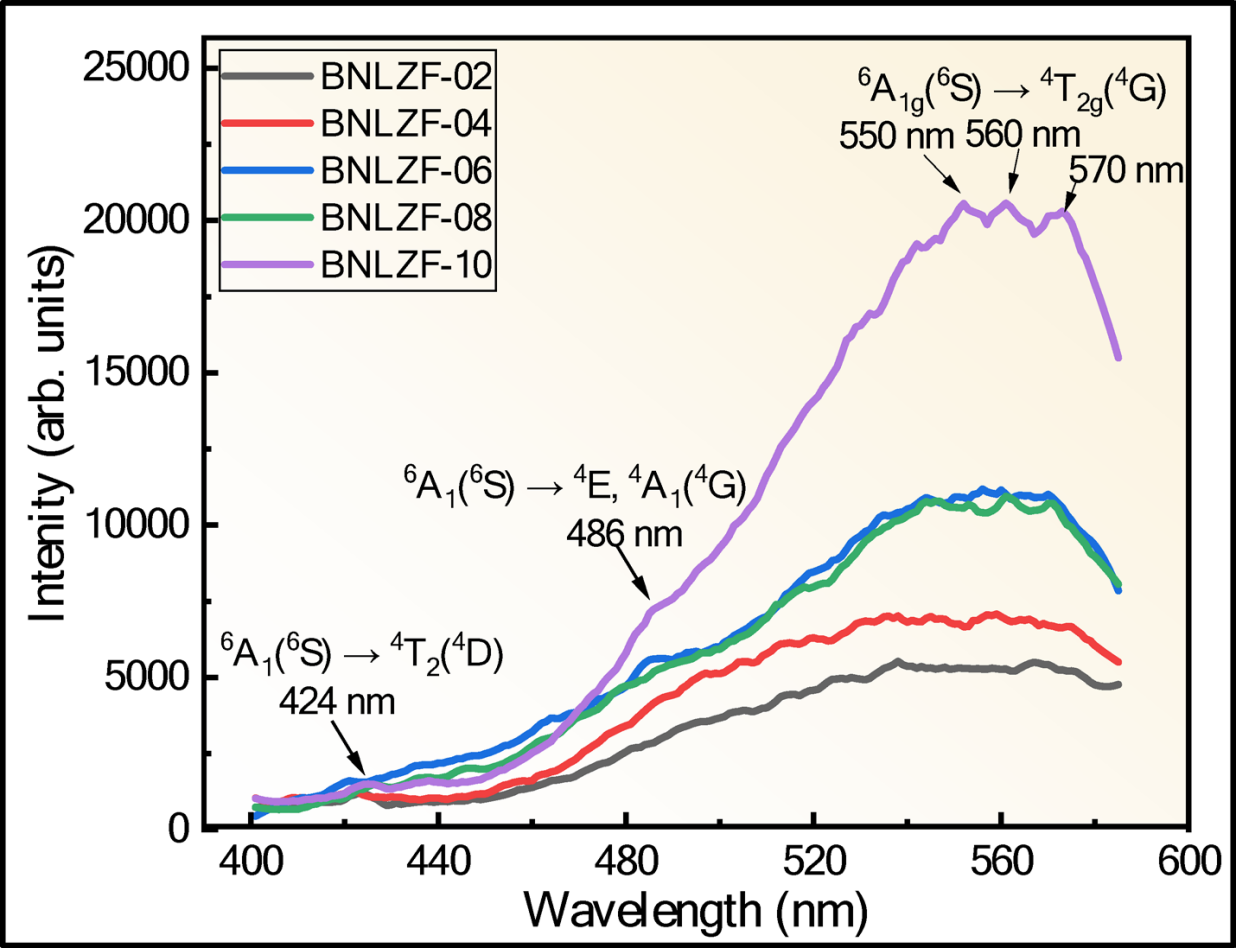
The excitation spectra (λ_emi_ = 598 nm) of BNLZF glass.

**Fig. 24:**
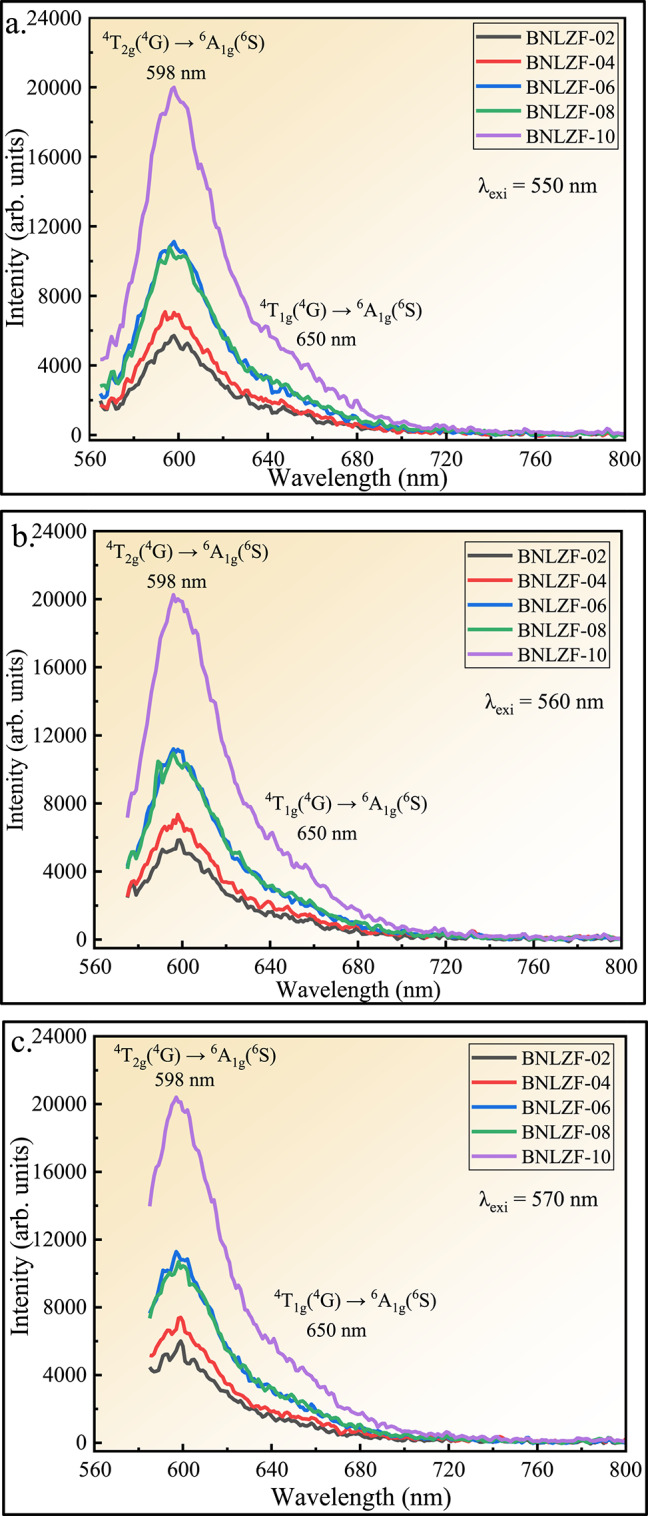
Emission spectra (**a**) λ_exi_ at 550 nm (**b**) λ_exi_ at 560 nm and (**c**) λ_exi_ at 570 nm of BNLZF glass.

**Fig. 25:**
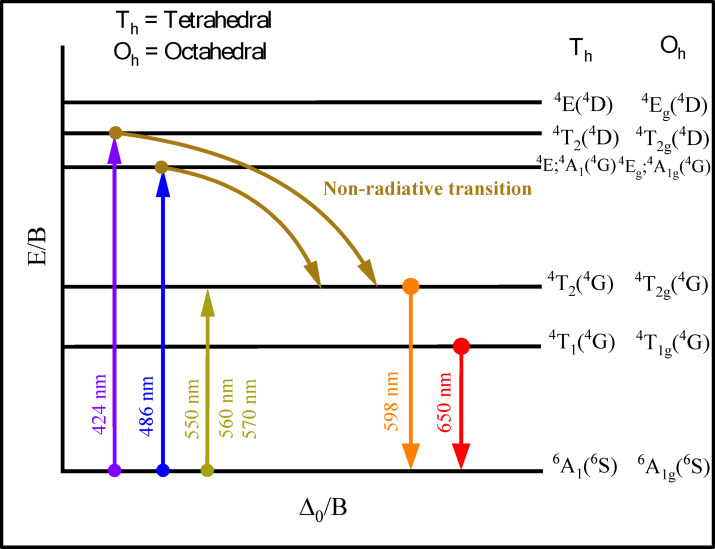
Schematic representation of the d^5^ energy level diagram.

Figures 23 and 24 show the excitation (λ_exi_) spectra and emission (λ_emi_) spectra of Fe^3+^ doped mixed alkali zirconia borate glasses. The PL spectra of BNLZF glasses arise due to the d^5^ configuration, which exhibits spin-forbidden *d*–*d* electronic transitions. The peaks in the excitation spectra, obtained at (1) 424 nm is due to the ^6^A_1_(^6^S) → ^4^T_2_(^4^D), (2) 486 nm is due to the ^6^A_1_(^6^S) → ^4^E,^4^A_1_(^4^G), and (3) 550 nm, 560 nm and 570 nm is due to the ^6^A_1g_(^6^S) → ^4^T_2g_(^4^G) transition^78,79^. The excitation is fixed at 550 nm, 560 nm and 570 nm, and the emission of a broad peak is observed at 598 nm is ascribed to the ^4^T_2g_(^4^G) → ^6^A_1g_(^6^S) and the shoulder peak at 650 nm is assigned to the ^4^T_1g_(^4^G) → ^6^A_1g_(^6^S) transition^79^. The intensity of the emission spectra increases as the Fe^3+^ concentration is increased in the BNLZF glass system by maintaining the orange-red emission at 598 nm. The increase in PL intensity is due to more Fe^3+^ ions absorbing photons at the excitation wavelength, creating a larger population of excited Fe^3+^ centres. Furthermore, the glasses do not exhibit concentration quenching within the investigated matrix range, suggesting the non-radiative mechanisms, like energy migration to quenching sites/cross-relaxation, are still confined. Therefore, both the preservation of an efficient radiative transition and the increasing number of Fe^3+^ centres are responsible for the increase in PL intensity. The schematic representation of the energy level diagram of the PL spectra is shown in Fig. 25. The orange-red colour emission at 598 nm leads to a prominent material for photonics applications.

**Fig. 26:**
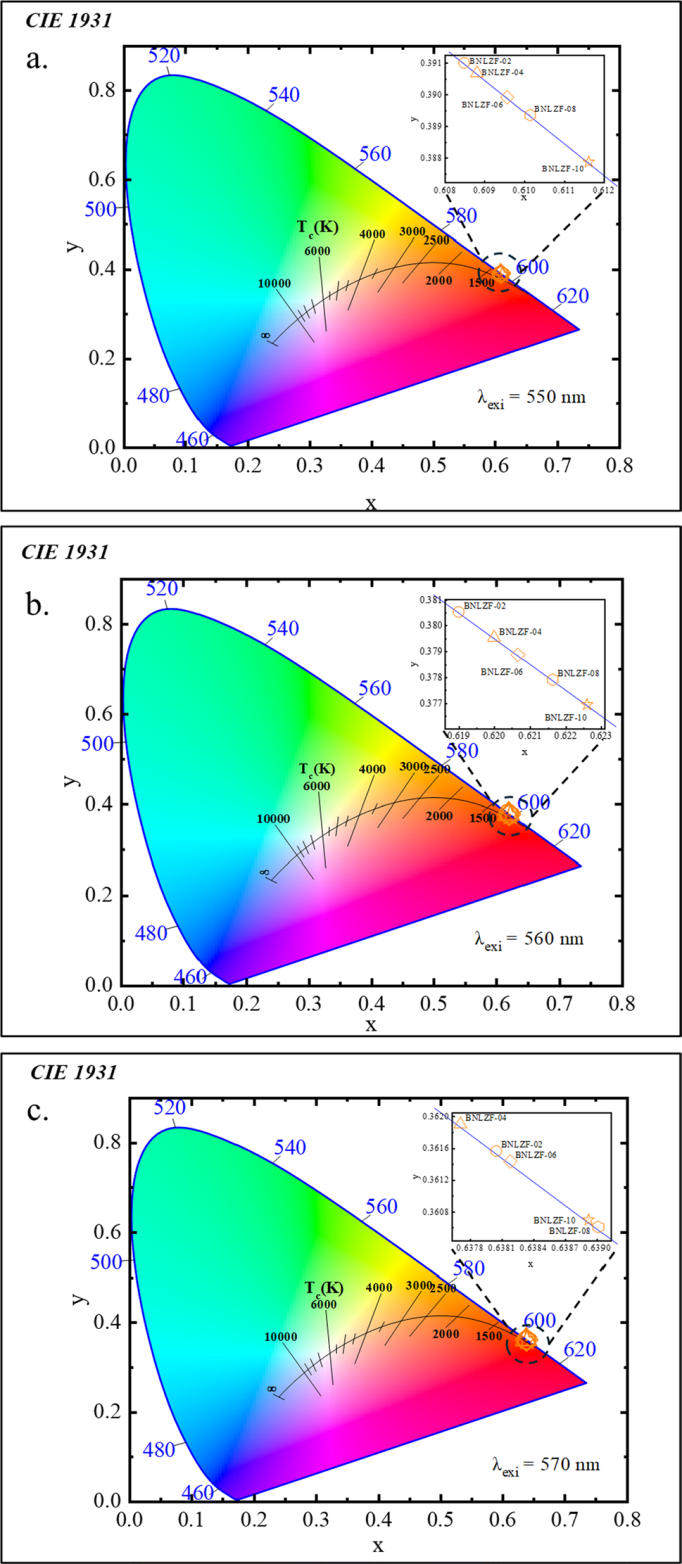
The CIE chromaticity diagram (**a**) λ_exi_ at 550 nm (**b**) λ_exi_ at 560 nm and (**c**) λ_exi_ at 570 nm of BNLZF glass.

The Commission Internationale de l’Éclairage (CIE) chromaticity diagram was utilised to assess the coordinates of the chromaticity of BNLZF glasses. The coordinates for synthesized BNLZF glasses are shown in Table 6, and they fall within the orange-red zone of the CIE diagram, as seen in Fig. 26. The CIE coordinates of BNLZF glasses meticulously resemble the coordinates (0.536, 0.444) of the renowned orange-red phosphor^80^. In accordance with this correlation, BNLZF glasses doped with Fe^3+^ exhibit pledge for orange-red photonic applications.

Additionally, the temperature of a Planckian blackbody radiator generating a colour most comparable to the observed source at the same brightness level was computed, which is known as the Correlated Colour Temperature (CCT). The CCT values were calculated using McCamy’s empirical formula^81,82^:

35 $$CCT = -449{m}^{3} + 3525{m}^{2} - 6823.3m +5520.33$$

where ($x, y$) are the coordinates determined from the CIE chromaticity diagram of the synthesized BNLZF glasses, $m=(x-{x}_{a})/(y-{y}_{a})$ is the inverse slope line, and ${x}_{a}$ and ${y}_{a}$ are the epicentres with the values of 0.332 and 0.186, respectively. The values of CCT for the prepared glass samples are listed in Table 7.

**Table 7:** The CIE coordinates and the CCT values of BNLZF glasses with error range ± 0.001.

Sample code	${\lambda }_{exi}$ (nm)	CIE coordinates		$m$	CCT (K)
		$x$	$y$		
BNLZF-02	550	0.6085	0.3910	1.3486	1628
BNLZF-04		0.6088	0.3907	1.3523	1629
BNLZF-06		0.6096	0.3899	1.3611	1631
BNLZF-08		0.6101	0.3894	1.3676	1633
BNLZF-10		0.6116	0.3879	1.3848	1639
BNLZF-02	560	0.6190	0.3805	1.4754	1684
BNLZF-04		0.6200	0.3795	1.4881	1693
BNLZF-06		0.6207	0.3789	1.4966	1699
BNLZF-08		0.6216	0.3779	1.5091	1708
BNLZF-10		0.6226	0.3770	1.5219	1718
BNLZF-02	570	0.6381	0.3616	1.7432	1959
BNLZF-04		0.6377	0.3619	1.7379	1952
BNLZF-06		0.6382	0.3614	1.7452	1962
BNLZF-08		0.6390	0.3606	1.7583	1980
BNLZF-10		0.6389	0.3607	1.7568	1978

At the λ_exi_ = 550 nm, the CCT values increased linearly from 1628 to 1639 K with the gradual increase in the Fe^3+^ concentration, showing a stable orange-red emission region. At λ_exi_ = 560 nm, CCT values increased from 1684 to 1718 K, indicating a steady shift in chromaticity coordinates to the reddish region. Conversely, at λ_exi_ = 570 nm, the CCT values fluctuated between 1972 and 1980 K, exhibiting a slightly non-linear dependence on Fe^3+^ concentration. This variation is attributed to changes in the Fe^3+^ site symmetry within the glass matrix in addition to local field effects in the glass network. These Fe^3+^ doped glasses exhibit low CCT values (below 2000 K) and emit warm light, making them suitable for laser active materials, optical amplifiers, and warm photonic applications^80,83^.

## Conclusion

The study on the BNLZF glass series was synthesised through the melt quenching technique.

1. XRD confirms the non-crystallinity by X-ray diffraction. Morphological and elemental analysis studies were seen through SEM images and EDS spectra, respectively.
2. The $\rho$ values were increased from 2.4197 to 2.4324 g cm^−1^, and ${V}_{m}$ values increased from 47.8548 to 49.2567 cm^3^ mol^−1^. Furthermore, the physical properties followed the same trend corresponding to the $\rho$ and ${V}_{m}$ values.
3. Deconvolution of the FTIR and Raman spectra explained the conversion of pentaborate and di-pentaborate to orthoborate units, verified with the decrease in N4 parameter value to 0.4952 from 0.5837 as the NBOs increased in the glass network.
4. The optical absorption band was observed at 450 nm, corresponding to the *d*–*d* electronic transition ^6^A_1g_ (^6^S) → ^4^A_1g_ (^4^G); ^4^E_g_ (^4^G). The redshift was observed from 300 to 387 nm as the concentration of Fe_2_O_3_ increased in BNLZF glass.
5. The direct optical bandgap decreased from 3.93 to 3.10 eV, and the indirect optical bandgap decreased from 3.44 to 2.77 eV. The Urbach energy increased from 0.245 to 0.273 eV, confirming the distortion in the glass system.
6. The $n$ values were increased from 1.54 to 1.57. The optical parameters were evaluated using the $n$ and the optical bandgap. The ${n}_{2}$ and ${\chi }^{(3)}$ values are found to be increased, and the values showed that BNLZF glasses were potentially suitable for nonlinear optical applications.
7. The PL spectra display orange-red emission centred at 598 nm attributed to the ^4^T_2g_(^4^G) → ^6^A_1g_(^6^S) transition, with a shoulder peak at 650 nm from the ^4^T_1g_(^4^G) → ^6^A_1g_(^6^S) transition. The excitation bands confirm efficient visible absorption from Fe^3+^ ions in mixed coordination sites. Increasing Fe^3+^ concentration enhances emission intensity while maintaining colour stability within the orangish-red region (CCT < 2000 K).

Overall, the findings show a strong relationship between structural modification and ensuring optical and luminescence properties, bridging the research gap and suggesting that the BNLZF glasses are promising materials for warm orange-red photonic applications.

## Data Availability

The data used and/or analysed during the current study are available from the corresponding author on reasonable request.
